# Targeting apoptotic pathways for cancer therapy

**DOI:** 10.1172/JCI179570

**Published:** 2024-07-15

**Authors:** Xiaobing Tian, Praveen R. Srinivasan, Vida Tajiknia, Ashley F. Sanchez Sevilla Uruchurtu, Attila A. Seyhan, Benedito A. Carneiro, Arielle De La Cruz, Maximilian Pinho-Schwermann, Andrew George, Shuai Zhao, Jillian Strandberg, Francesca Di Cristofano, Shengliang Zhang, Lanlan Zhou, Alexander G. Raufi, Arunasalam Navaraj, Yiqun Zhang, Nataliia Verovkina, Maryam Ghandali, Dinara Ryspayeva, Wafik S. El-Deiry

**Affiliations:** 1 Laboratory of Translational Oncology and Experimental Cancer Therapeutics and; 2 Department of Pathology and Laboratory Medicine, Warren Alpert Medical School, Brown University, Providence, Rhode Island, USA.; 3 Joint Program in Cancer Biology, Lifespan Health System and Brown University, Providence, Rhode Island, USA.; 4 Legorreta Cancer Center at Brown University, Providence, Rhode Island, USA.; 5 Pathobiology Graduate Program, Brown University, Providence, Rhode Island, USA.; 6 Hematology/Oncology Division, Department of Medicine, Lifespan Health System and Brown University, Providence, Rhode Island, USA.

## Abstract

Apoptosis is a form of programmed cell death that is mediated by intrinsic and extrinsic pathways. Dysregulation of and resistance to cell death are hallmarks of cancer. For over three decades, the development of therapies to promote treatment of cancer by inducing various cell death modalities, including apoptosis, has been a main goal of clinical oncology. Apoptosis pathways also interact with other signaling mechanisms, such as the p53 signaling pathway and the integrated stress response (ISR) pathway. In addition to agents directly targeting the intrinsic and extrinsic pathway components, anticancer drugs that target the p53 and ISR signaling pathways are actively being developed. In this Review, we discuss selected and promising anticancer therapies in various stages of development, including drug targets, mechanisms, and resistance to related treatments, focusing especially on B cell lymphoma 2 (BCL-2) inhibitors, TRAIL analogues, DR5 antibodies, and strategies that target p53, mutant p53, and the ISR.

## Introduction

Apoptosis is a form of regulated cell death with a critical role in development and homeostasis (1). Activation of apoptotic pathways results in destruction of target cells with minimal inflammatory response and disruption to surrounding tissue. Preventing cancer is an important function of apoptosis (2), and dysregulation and evasion of apoptosis are hallmarks of cancer. Tumor cells employ multiple mechanisms to evade apoptosis, including expression of apoptosis inhibitors as a means of acquiring resistance to cancer therapies. Much effort has gone into developing drugs to reinstate or promote apoptosis in cancer cells. Below, we will briefly describe the major apoptotic pathways, then highlight major advancements toward targeting these pathways and other regulators of apoptosis in cancer cells.

## Intrinsic and extrinsic apoptotic pathways

**Figure 1:**
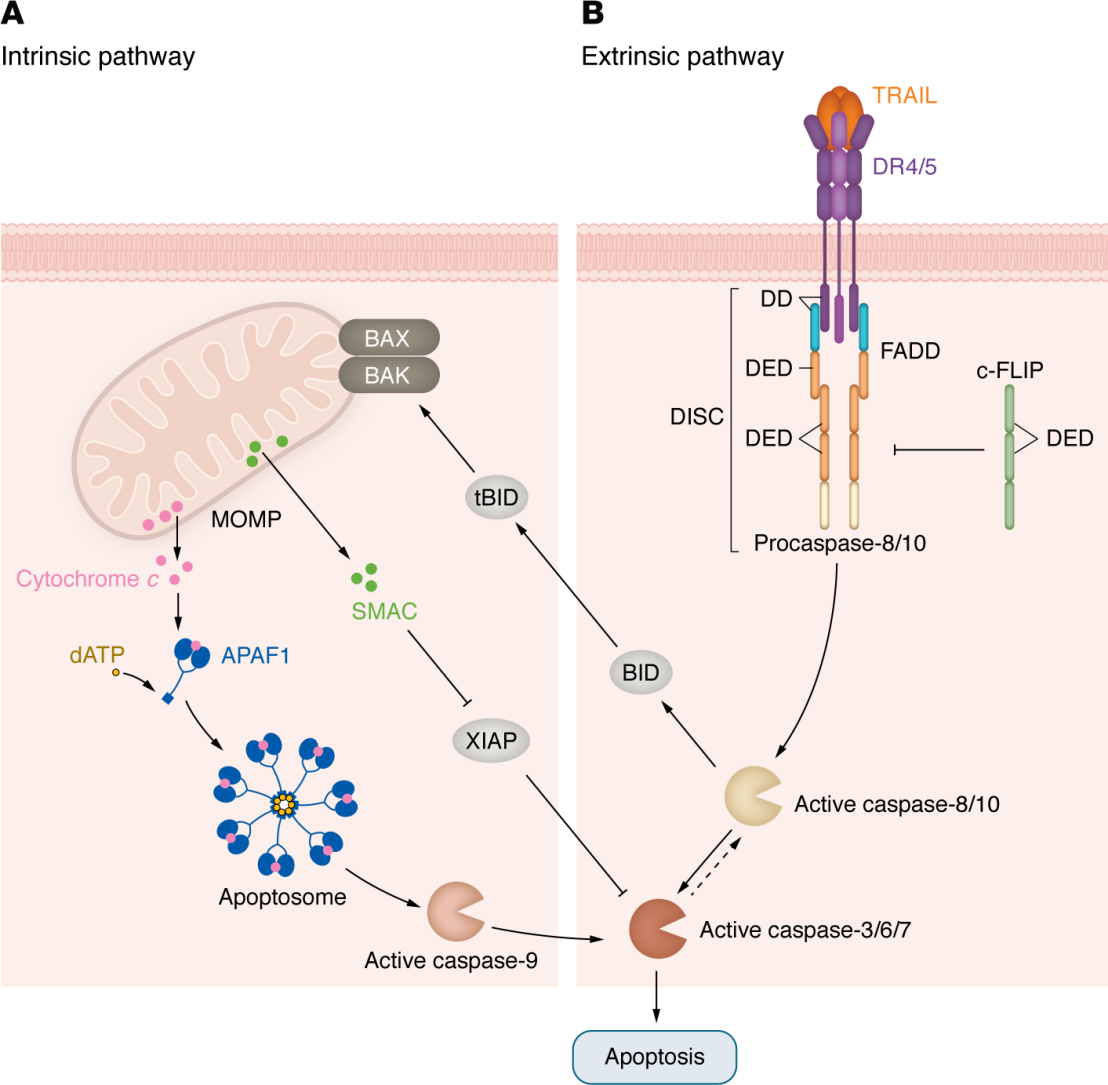
Intrinsic and extrinsic apoptosis pathways. (**A**) Intrinsic apoptosis pathways. Upon activation, BAK and BAX undergo conformational changes and oligomerization, forming pores in the MOM and causing irreversible MOM permeabilization (MOMP), the critical step for intrinsic apoptosis (3), allowing release of cytochrome *c* and SMAC. Cytochrome *c* and dATP join APAF-1 and the initiator protein procaspase-9 to form the apoptosome, while SMAC interacts with IAPs (see below). Within the apoptosome, procaspase-9 is cleaved into active caspase-9, which cleaves and activates the apoptosis effector proteins caspase-3, -6, and -7 (3). (**B**) Extrinsic apoptosis pathway. Upon ligand binding, DR4 and DR5 trimerize and aggregate within the cell membrane, a process known as capping. This is followed by recruitment of the adaptor protein FADD, which has a death effector domain (DED). Initiator procaspase-8 and -10 also have DEDs that bind to FADD at its DED, forming the DISC. Procaspase-8 and -10 are activated within the DISC and in turn cleave and activate executioner caspase-3, -6, and -7. Activation of procaspase-8/10 is negatively regulated by c-FLIP. c-FLIP competes directly with procaspase-8 for binding to FADD through homotypic DED interactions, thus inhibiting procaspase-8 recruitment and activation at the DISC (9-12). Activated caspase-8 also cleaves the BH3 subfamily member BID to active form truncated-BID (tBID). tBID translocates to the MOM and initiates apoptosis through its interactions with proapoptotic effector proteins BAK and BAX. BID cleavage and translocation to the mitochondria link the extrinsic cell death pathway to the intrinsic apoptotic pathway and amplify the apoptotic response. This amplification mechanism is required for effective apoptosis in certain cells, denoted as type II cells for their mechanism of apoptosis, in contrast with type I cells, which undergo extrinsic apoptosis independently of intrinsic apoptosis pathway induction (13, 14).

Two pathways are considered the major drivers of apoptosis in all cells: the intrinsic pathway, initiated by the formation of Bax and Bak pores on the mitochondrial outer membrane (MOM), and the extrinsic pathway, triggered by death receptors (DRs) on the plasma membrane (Figure 1).

### Intrinsic apoptosis

**Figure 2:**
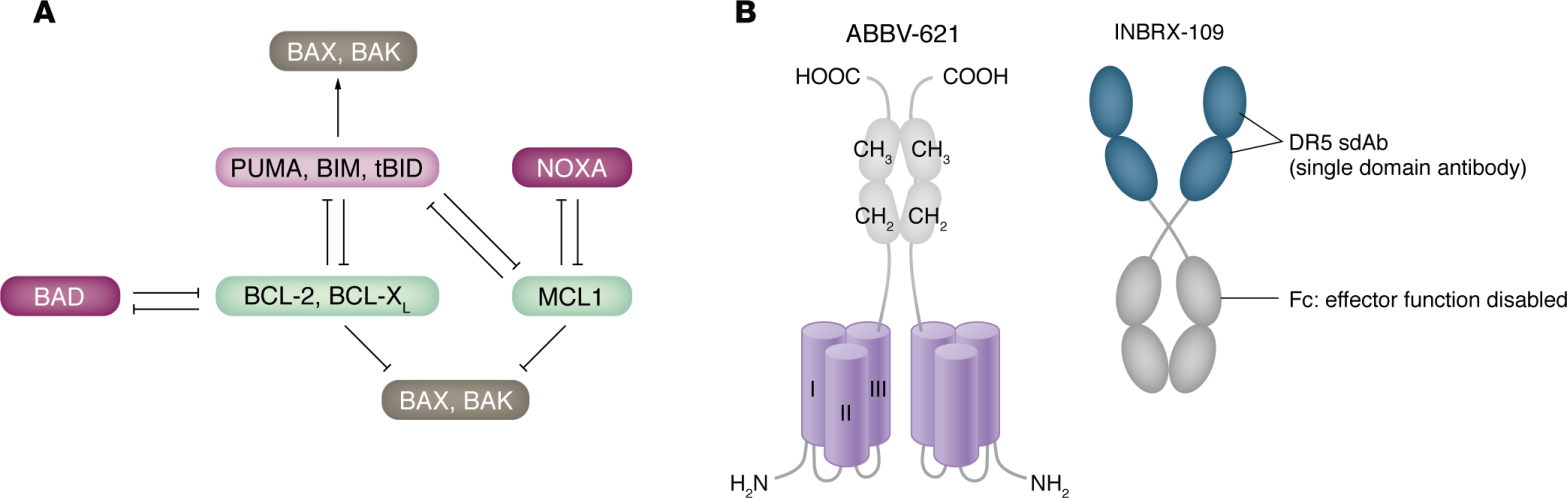
Targets in the intrinsic and extrinsic apoptosis pathways. (**A**) Interactions of the BCL-2 protein family. The multi-BH domain family members either suppress apoptosis (e.g., BCL-2, BCL-XL, and MCL-1) or promote apoptosis (e.g., BAX, BAK), whereas the BH3-only subfamily members identified to date (e.g., BAD, BID, PUMA, NOXA, and BIM) function exclusively to promote cell death (3, 4) BH3-only proteins can be divided into activators or sensitizers. The activators PUMA, tBID, and BIM directly activate BAK and BAX and interact with antiapoptotic proteins to promote MOMP (5, 6). In contrast, the sensitizers BAD and NOXA only interact with the antiapoptotic proteins and do not activate BAK and BAX (7, 8). Interactions with antiapoptotic BCL-2 proteins and activator BH3-only proteins regulate BAK and BAX activity. (**B**) High-potency TRAIL receptor agonists. ABBV-621 is a hexavalent TRAIL fusion protein with Fc-FcγR interactions disabled by IgG Fc D297S mutation. INBRX-109 is a tetravalent DR5 agonistic antibody with Fc effector function disabled by forming a cycle.

In most mammalian cells, the B cell lymphoma 2 (BCL-2) protein family regulates the intrinsic pathway (Figure 1A) (3). BCL-2 family members are characterized by the presence of up to four distinct segments of amino acid homology, termed BCL-2 homology (BH) domains. The interactions of the BCL-2 protein family are depicted in detail in Figure 2A (3–8).

### Extrinsic apoptosis

Perturbations of the extracellular microenvironment that trigger release of Fas-L, TNF, and TRAIL activate the extrinsic apoptotic pathway when these extracellular ligands bind to Fas, TNF receptors, and DR4/5, respectively. As ongoing efforts in anticancer drug discovery and development continue to focus on targeting DR4/5, we will focus on their role in apoptosis here. The mechanism of DR4/5 activation is summarized in Figure 1B (9–14).

### IAPs and execution of apoptosis

Inhibitors of apoptosis (IAPs) constitute a highly conserved family of proteins defined by the presence of 1–3 protein motifs called baculovirus IAP repeats (BIRs). Most BIRs form a surface hydrophobic groove that specifically binds a conserved tetrapeptide motif, called IAP-binding motif (IBM), found in the active subunits of caspase-3, -7, and -9 (15). Second mitochondrial activator of caspase (SMAC) released by MOM permeabilization blocks IAPs (including XIAP) to promote cell death (16) (Figure 1A). Caspases-3, -6, and -7 execute apoptosis via the proteolysis of thousands of cellular proteins. The main features of cells undergoing apoptosis include chromatin condensation, DNA fragmentation, membrane blebbing, and cytoskeletal rearrangement (4).

## Targeting intrinsic apoptosis in cancer therapy

Cancer cells resist apoptosis using a variety of mechanisms. Defects in intrinsic pathways include the following: (a) acquiring of caspase gene mutations that inhibit caspase function (2); (b) overexpression of antiapoptotic BCL-2 family proteins (2, 15); (c) overexpression of IAPs (17); (d) loss and inactivation of apoptotic effectors BAX and BAK (2, 18); (e) insufficient release of cytochrome *c* and mutation of lysine residues (especially K72) of cytochrome *c* that abrogate the apoptosome formation, causing inhibition of caspase activation (19, 20); and (f) defects in extrinsic pathway signaling. These defects include (a) overexpression of apoptosis-inhibiting decoy receptors (e.g., DcR1/2), which compete with DR4/5 for TRAIL binding (21); (b) decreased DR4/5 activity; and (c) death-inducing signaling complex (DISC) inhibition by FLICE-like inhibitory protein (c-FLIP) (22). For instance, colorectal cancer (CRC) cells have decreased activity of DR4/5 that contributes to their resistance to apoptosis (21, 23). Decreased expression of DR4/5 seems to result from defective p53, impaired transport from ribosomes, defective redistribution of DR4/5 in lipid rafts and mutations, epigenetic changes (23, 24), or overexpression of DcR3.

Tumor cells can overexpress multiple inhibitors of both apoptotic pathways, including in the process of acquiring resistance to cancer therapy. Upregulation of the antiapoptotic BCL-2 family proteins and decreased expression of proapoptotic proteins are responsible for cancer cell resistance to chemotherapy and radiation. For example, BCL-2 gene expression is elevated in over half of all cancers and XIAP is overexpressed in many tumors (2, 4, 17).

Recent development of apoptosis-targeted drugs has focused on the intrinsic pathway, including BCL-2, MCl-1, and IAP inhibitors (25). In this Review, we focus our discussion on BCL-2–specific inhibitors due to the relatively recent approval of the BCL-2 inhibitor venetoclax by the US FDA.

### Venetoclax

BCL-2 inhibitors, also known as BH3 mimetics, are among the frontrunners of agents that were developed as a targeted approach to directly alter the intrinsic apoptosis pathway. BH3 mimetics are small molecules that mimic the binding of BH3-only proteins to the hydrophobic pockets within antiapoptotic BCL-2 proteins. In 2016, venetoclax (ABT-199) was the first agent targeting BCL-2 to be approved by the US FDA for the treatment of patients with chronic lymphocytic leukemia (CLL) harboring 17p deletion. Venetoclax binds to BCL-2, leading to the release of BIM, which in turn directly activates BAX and BAK (26–28) (Table 1 and Figure 2A). In May 2019, venetoclax was approved by the FDA for the frontline treatment of patients with CLL owing to the superior efficacy of venetoclax plus the anti-CD20 antibody obinutuzumab over chlorambucil plus obinutuzumab, thus providing a chemotherapy-free option for CLL patients (29). Venetoclax was also approved by the FDA in 2020 for the treatment of elderly patients with newly diagnosed acute myeloid leukemia (AML) who are ineligible for standard induction chemotherapy (i.e., >75 years old) (30, 31).

## Targeting extrinsic apoptosis in cancer therapy

### TRAIL analogs

TRAIL is a transmembrane trimeric glycoprotein that can be cleaved by metalloproteinases and released as a soluble factor (32, 33). Both soluble and membrane-bound forms of TRAIL can bind to DR4/5, triggering the extrinsic apoptosis pathway (Figure 1A). TRAIL signaling selectively induces cancer cell apoptosis without causing toxicity to normal cells. Based on this unique activity profile, many agents targeting this pathway, including recombinant human TRAIL (rhTRAIL, or dulanermin) and DR4/5 agonist antibodies (lexatumumab and conatumumab for DR5, mapatumumab for DR4), have been developed and evaluated in vitro and in vivo (34–37). Preclinical data indicated that both classes of molecules are generally well tolerated, but ultimately, they showed limited anticancer activity in patients. One factor contributing to limited anticancer activity is rhTRAIL’s very short half-life in blood, from 0.56 to 1.02 hours (38, 39). Although rhTRAIL induces trimerization (also known as lower-order trimerization) of DR4/5, its soluble form of TRAIL has limited capacity to induce high-order clustering of the DR trimers, resulting in a weak apoptotic signal (40). For DR4/5 agonist antibodies, lower-order receptor trimerization is a major limitation due to the bivalent structure of the antibodies (40, 41).

#### TLY012.

Second-generation rhTRAIL therapeutics were developed to address the clinical limitations of previous TRAIL or TRAIL receptor agonist antibodies. One such conjugate is TLY012, where attaching polyethylene glycol (PEG) to the N-terminus of rhTRAIL increases its size, thereby reducing its clearance by renal filtration (Table 1). This modification prolongs the half-life of TLY012 to 12 to 18 hours, resulting in greater antitumor effect both in vitro and in vivo in CRC models (42). TLY012 also had marked activity against fibrotic cells, characterized by increased expression of DR5 (43). These results supported the orphan drug designation by the FDA in 2019 for the treatment of systemic sclerosis.

Pancreatic cancer cells are notoriously resistant to extrinsic TRAIL-induced apoptosis and undergo type II extrinsic apoptosis (44, 45). TRAIL resistance in pancreatic cancer cells occurs partially due to their overexpression of various IAP family proteins (e.g., cIAP-1, XIAP, and survivin) that block the cleavage of caspase-3, -7, or -9 (46) (Figure 1A). cFLIP blocks TRAIL-induced caspase-8 activation by competing with caspase-8 for binding to Fas-associated death domain (FADD) (25). To this end, ONC201 is a TRAIL- and DR5-inducing compound that may help overcome resistance to TRAIL-induced apoptosis. The combination of ONC201 and TLY012 can induce selective, synergistic apoptosis in six pancreatic cancer cell lines and significantly delays tumor xenograft growth in vivo (47). The combination of TLY012 and PD-1 immune checkpoint inhibition also reduced the growth of pancreatic tumors in vivo and promoted tumor infiltration of CD8^+^ T cells, suggesting the potential of TLY012 to enhance the effects of checkpoint inhibitors (48).

#### Eftozanermin alfa (ABBV-621).

In clinical studies of TRAIL derivatives and DR4/5 agonists, overall response rates did not meet the requirements to pass clinical trials (36, 38, 39, 49–54). DR4/5 agonist antibodies show potent antitumor effects in various tumor xenograft models, but not in clinical trials. There are two reasons: (i) rhTRAIL, TRAIL derivatives, and DR4/5 agonist antibodies do not induce high-order receptor clustering, which reduces apoptosis signaling; and (ii) IgG can cross-link Fc region of the antibodies to Fcγ receptors (FcγRs). Mouse models have lower IgG levels than do cancer patients. In patients, high concentrations of endogenous IgG compete for FcγR binding, inhibiting efficient clustering of agonistic antibodies (55, 56).

To address this problem, APG350 was engineered to potently increase receptor clustering for full antitumor activity independently of FcγR. It contains two single-chain TRAIL receptor–binding domains (scTRAIL-RBD), and each scTRAIL-RBD carries three binding sites for a receptor, resulting in a dimer with six binding sites for DR4/5 (Figure 2B). This structural change improves low-order clustering ability and leads to induction of efficient high-order clustering (55, 57). Modification of APG350 yielded eftozanermin alfa by introducing a mutation into the IgG1-Fc binding site and eliminating Fc-FcγR interaction (Figure 2B). Eftozanermin alfa shows highly specific and strong binding to death receptors and induces optimal receptor clustering, leading to on-target apoptosis in vitro and in vivo. Also, clinical trials show that eftozanermin alfa has fewer side effects and is well tolerated in patients with various cancers (57, 58).

In the 105 patients with advanced solid tumors who were studied, eftozanermin alfa monotherapy led to tumor responses in two patients with CRC and one with pancreatic cancer (58). The combination of eftozanermin alfa and venetoclax was investigated in patients with refractory AML and showed an encouraging response rate of 30%, including four complete responses (58). Eftozanermin alfa fully occupies the binding sites on the death receptors, leading to enhanced cleavage of PARP and apoptosis. Importantly, the combination treatment also induced more tumor infiltration of immune cells, such as CD4^+^ T cells, in treated tumor specimens. Despite these encouraging results demonstrating target engagement and signal of clinical activity, the only active clinical trial with eftozanermin listed at ClincalTrials.gov is a phase II trial investigating eftozanermin alfa plus bortezomib and dexamethasone for patients with multiple myeloma (MM) (NCT04570631) (Table 1). 

#### Agonistic DR5 antibodies

##### TAS266.

Nanobodies are small, single-domain antibody fragments derived from camelids that have a high affinity for their target antigen (59). TAS266 is an agonistic tetravalent nanobody targeting DR5 consisting of four identical humanized VHH antibody fragments connected through three linkers. TAS266 specifically binds to and activates DR5 receptors, leading to apoptosis. It is more potent than cross-linked DR5 antibodies or TRAIL for inducing cell apoptosis (41). In vivo, TAS266 elicited single-dose tumor regressions in multiple human tumor xenograft models (59). However, in a phase I clinical trial, TAS266 showed severe hepatotoxicity that was attributed to hyperclustering by preexisting antidrug antibodies (ADAs), leading to suspension of the clinical trial and development of this drug (41).

##### INBRX-109.

INBRX-109 is a third-generation, tetravalent agonistic antibody engineered to reduce the hepatotoxicity based on a single domain antibody platform (Figure 2B). It consists of two identical camelid heavy chain–only antibody-binding domains targeting DR5. These domains are joined end to end with an effector-silenced Fc constant domain based on human immunoglobulin G1. INBRX-109’s design eliminates recognition by preexisting ADAs (41). In a phase I study, INBRX-109 showed antitumor activity in patients with chondrosarcoma, a rare bone cancer, resulting in a disease control rate of 87% among 31 patients. Two patients had tumor partial responses, a rare positive outcome with this tumor type, which is resistant to chemotherapy and radiation therapy, and 25 patients had stable disease (60). The treatment was well tolerated, with low grade liver-related adverse events. These results led to an ongoing randomized phase II trial of INBRX-109 in conventional chondrosarcoma (NCT04950075). In 2021, the FDA granted fast-track designation to INBRX-109 for the treatment of patients with unresectable or metastatic chondrosarcoma (Table 1).

## Targeting p53 and mutant p53 in cancer therapy

**Figure 3:**
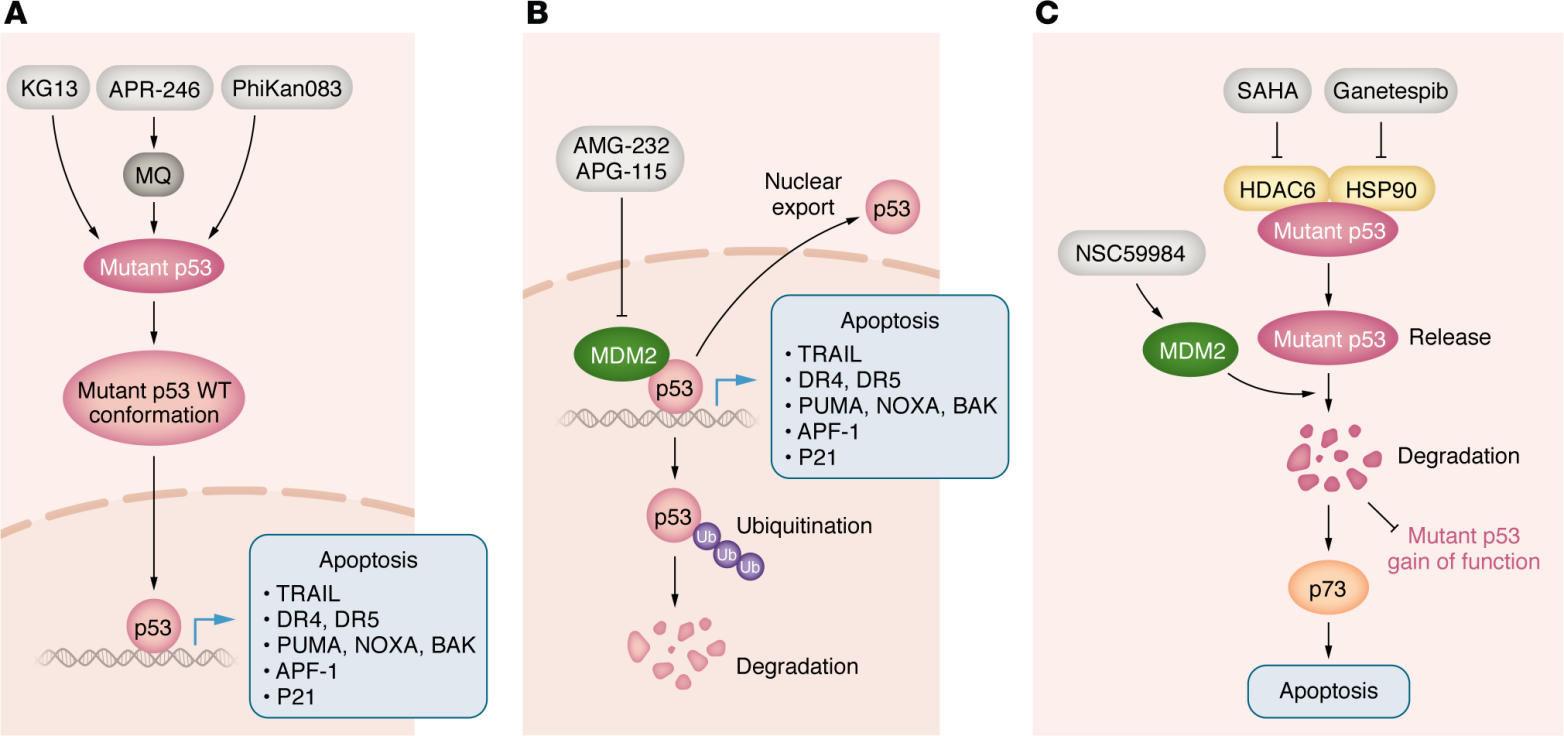
Strategies targeting p53 and mutant p53. (**A**) Reactivation of mutant p53. Direct binding of a small molecule (gray boxes) to a mutant p53 promotes and stabilizes WT p53 folding and conformation, leading to restoration of specific DNA binding and transcription of p53 target genes. This will induce tumor cell apoptosis or senescence. (**B**) Inhibition of MDM2. MDM2 binds to p53 directly through its N-termini and inhibits p53 function through two major mechanisms: (a) upon binding, MDM2 ubiquitinates p53, promoting proteasomal degradation of p53; (b) MDM2 promotes export of p53 out of the cell nucleus. (**C**) Depletion of mutant p53. Small molecules inhibit MTp53 gain-of-function and dominant-negative effects through degradation of MTp53.

p53 is the guardian of the genome and an important upstream regulator of apoptosis and other key biological functions (61). The essential growth-arrest and proapoptotic genes induced by activated p53 include *CDKN1A* (p21), *PUMA*, *NOXA*, *BAK*, apoptotic protease-activating factor-1 (*APAF-1*), *TRAIL*, and *DR5* (62–66) (Figure 3). Therefore, p53 affects both intrinsic and extrinsic apoptosis pathways. p53 is inactivated in around 50% of human cancers and in almost all tumor cell lines in culture (67). Two important mechanisms responsible for inactivation of p53 include mutation of the *TP53* gene and negative regulation of WT p53 protein by MDM2. DNA-damaging drugs can potently activate WT p53; however, secondary malignancies due to increased mutation burden remain a substantial concern (68). Restoration of the p53-regulated transcriptome without DNA damage represents an important anticancer strategy. Approaches using this strategy can be divided into three categories. The first approach uses agents targeting p53-negative regulators to activate WT p53, such as MDM2 inhibitors (69, 70). The second approach involves directly targeting mutant p53 by small molecules to restore its conformation and WT p53 function (71–73). The third approach is indirect and bypasses p53 by compounds that upregulate proapoptotic p53 targets in p53-deficient tumors via inducing the integrated stress response (ISR) (74, 75) or activating p73 (76).

### Reactivation of suppressed WT p53

#### MDM2 inhibitors.

MDM2 is a nuclear-localized E3 ligase, and its overexpression is common in various cancers. MDM2 binds to and ubiquitinates p53, causing p53 proteasomal degradation and promoting export of p53 out of the cell nucleus (77). In addition, *MDM2* is a p53 target gene and inhibits p53 activity through a feedback mechanism (78) (Figure 3). MDM2 inhibitors bind to the p53-binding pocket in MDM2 and inhibit p53/MDM2 interaction, leading to stabilization of p53 and induction of p53-dependent cell-cycle arrest or apoptosis. The first MDM2 inhibitors identified were nutlins, including nutlin-3a and idasanutlin. Idasanutlin clinical trials were terminated due to futility (NCT03287245 and NCT02545283). Later, other classes of MDM2 inhibitors were developed (79), such as AMG-232 (80), siremadlin (81), and alrizomadlin (APG-115) (82) (Figure 3 and Table 2). In AML models, APG-115 treatment leads to a reduction in tumor burden and an improvement in survival period. Azacitidine, decitabine, and cytarabine can cause DNA damage and activate p53. Combining APG-115 with these standard treatment drugs demonstrated synergistic effects, which are possibly due to enhanced activation of the p53/p21 pathway (83). A phase 2 clinical trial has been launched to evaluate APG-115 in combination with PD-1 antibody pembrolizumab in patients with solid tumors, including those with *TP53-*mutant tumors (82) (NCT03611868). The clinical trial showed that side effects did not overlap between the 2 drugs, the combination was well tolerated in patients, and APG-115 sensitized the antitumor activity of pembrolizumab. In September 2021, the FDA granted fast-track designation to APG-115 for the treatment of patients with unresectable or metastatic melanoma that is either relapsed or refractory to previous immunotherapy agents. Clinical trials testing the efficacy of MDM2 inhibitors and combination treatments are still ongoing, and the results are yet to be seen.

Although MDM2 is best known for its role in p53 inactivation, this protein also shows p53-independent functions. These include ubiquitination of other proteins (including androgen receptor and transcriptional factor HBP1), regulation of transcription, participation in DNA repair, and regulation of mitochondrial respiration (78, 84–86).

### Restoration of mutant p53 function

#### Eprenetapopt (APR-246).

While “boosting WT p53” is a good strategy, it is not applicable to p53-mutated and p53-deleted tumors. Approximately 50%–60% of human cancers carry mutations in the p53 gene (87). The mutations can lead to a loss of normal p53 tumor-suppressor functions and acquisition of new, cancer-promoting functions. These mutant p53 proteins can promote cancer cell proliferation, migration, and invasion, as well as contributing to genetic instability and drug resistance (87, 88). Restoring the p53 signaling pathway involves various strategies (87). Eprenetapopt binds to a mutant p53 and induces a conformational change of a mutant p53 protein, leading to WT-p53 conformation and restoring WT function to mutant p53 (87–89).

Eprenetapopt was widely investigated and advanced all the way to phase III trials (89–91). Once eprenetapopt enters cells, it is converted to the reactive electrophile methylene quinuclidinone (MQ), which binds covalently to the p53 core domain (Figure 3). Cys277 is a prime binding site for MQ in p53 and is essential for MQ-mediated thermostabilization of R175H- and R273H-mutant p53, converting the protein to a WT p53–like conformation and exhibiting WT p53 activity. Importantly, both Cys124 and Cys277 are required for eprenetapopt-mediated R175H-mutant p53 reactivation (89–91).

In addition, eprenetapopt has been shown to have alternative mechanisms to induce cell death, such as eprenetapopt’s reaction with other thiol group–containing cellular molecules. Thus, eprenetapopt has been reported to attach to and deplete thiol-containing GSH, resulting in increased ROS (92–95). The ability of eprenetapopt to increase ROS levels may contribute to anticancer activity observed in WT p53 and p53-depleted cancer cells (71, 90, 96, 97). Along with the ability to reactivate mutant p53 and generate ROS, eprenetapopt exhibited potent antitumor activity in a wide range of preclinical cancer models in vitro and in vivo (71, 90, 96).

A phase Ib/II study of the combination of eprenetapopt and AZA in 45 patients with *TP53*-mutant myelodysplastic syndromes or AML showed a favorable toxicity profile and led to clinical responses in 71% of patients, including complete responses in 44%. However, the combination of eprenetapopt plus AZA failed to significantly increase the rate of complete responses in a phase III trial in *TP53*-mutant myelodysplastic syndromes, ending the clinical development of this drug (98) (Table 2).

#### KG13.

Besides mutations on the DNA-binding domain (DBD) of p53, Y220C is the most common cancerous mutation and is responsible for approximately 100,000 cancer cases per year worldwide (99). It creates a cavity on the surface of p53 and also leads to loss of DNA-binding ability at room temperature (72). The small molecule PhiKan083 is a lead compound that is able to insert into the Y220C pocket (100, 101) (Figure 2). Although the PhiKan compounds have demonstrated the potential to target p53 Y220C, none of them satisfy the potency requirements of drug candidates because PhiKan are reversible binding compounds (72). KG13, an azaindole derivative (72), selectively and covalently attaches to the cysteine of mutant p53 Y220C. In Guiley and Shokat’s initial characterization of this small molecule, KG13 restored WT p53 thermal stability of the mutant p53 (Figure 3). KG13 restored p53 Y220C DNA-binding ability and led to the expression of proapoptotic p53 target genes, holding potential as a specific therapy for p53 Y220C-mutated cancers (72). 

### Novel compounds causing depletion of mutant p53

Depletion of mutant p53 prevents both mutant p53 gain-of-function and dominant-negative effects. HSP90 is an ATP-dependent molecular chaperone that reversibly binds to and stabilizes p53. Ganetespib binds to the ATP-binding domain of HSP90, inhibiting the ATPase activity of the HSP90 core protein (102, 103). Ganetespib potently inhibited cancer cell proliferation in vitro and in human tumor xenografts in multiple types of cancer (102–105). However, these studies did not address whether ganetespib’s effects are relevant to WT or mutant p53. SAHA (vorinostat) is an FDA-approved inhibitor of class I, II, and IV histone deacetylases (HDACs) and epigenetically regulates the malignant properties of multiple cancer types (106). Mutant p53s are stabilized by forming an HDAC6/HSP90/mutant p53 complex in cancer cells (107–110) (Figure 3). Alexandrova et al. reported that genetic and pharmacological depletion of mutant p53 (R248Q) by ganetespib or SAHA inhibits the growth of human breast MDA-MB-231 cancer cells in a mutant p53-dependent manner (107–109). In p53^R172H/R172H^ and p53^R248Q/–^ mice, ganetespib treatment inhibited tumor growth and extended survival, which was not observed in control p53^–/–^ mice (107). Ganetespib was investigated in phase I/II clinical trials in combination with paclitaxel for the treatment of p53-mutated platinum-resistant ovarian cancers, and it did not improve patient outcomes (111). Despite negative results in ovarian cancer, the clinical activity of ganetespib in other p53-mutated tumors as monotherapy or in combination with other agents remains unknown. Zhang et al. reported that compound NSC59984 induces mutant p53 degradation through activation of MDM2 and stimulates p73 activity, leading to p73-mediated cell apoptosis in p53-mutated CRC cells (76) (Figure 3).

## Targeting the ISR in cancer therapy

**Figure 4:**
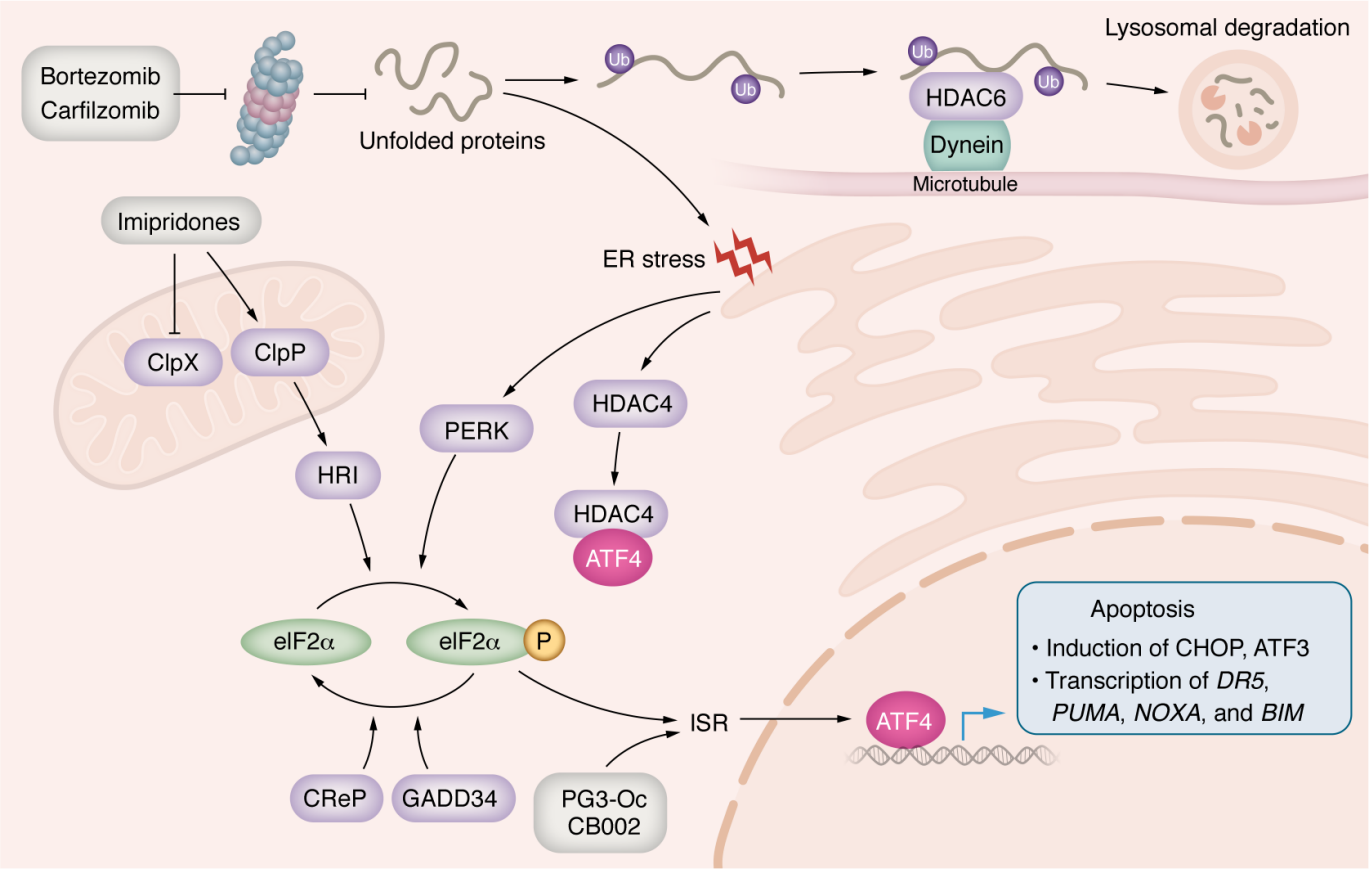
Targeting the ISR and overcoming resistance mechanisms. From top left: in the cell death pathway of the ISR, ATF4 induction can be achieved by eIF2α kinase activators, such as bortezomib, carfilzomib, and imipridones (gray boxes). ATF4 directly or indirectly through the induction of transcriptional factors CHOH or ATF3 regulates the expression of proapoptotic genes, such as DR5, PUMA, NOXA and BIM, which promotes cell apoptosis (lower right). Resistance mechanisms include movement of the PUP-HDAC6-dynein complex to aggresome along the microtubule (upper right). The aggresome is ultimately degraded in lysosomes. Additionally, ER stress induced by the proteasome inhibitors can also promote HDAC4 binding to ATF4 to prevent its nuclear translocation and inhibit ATF4 transcriptional activity.

ISR is a conserved signaling pathway in eukaryotic cells that is activated in response to a range of physiological changes and different pathological conditions. The ISR is triggered by eIF2α phosphorylation that is mediated by the eIF2α kinases PERK, GCN2, HRI, and PKR. The kinases are activated by different stressors, such as ER stress (PERK), amino acid deficiency (GCN2), heme decrease (HRI), and dsRNA virus infection (PKR) (112). We will focus on proteasome inhibitors and imipridones, which activate PERK and HRI, respectively (Figure 4). eIF2α phosphorylation inhibits 5′-cap–dependent protein translation, which selectively activates the translation of mRNAs with uORFs in the 5′-UTR region, such as ATF4 (112, 113), a basic leucine zipper (bZIP) transcription factor belonging to the ATF/CREB family (113). ATF4 regulates expression of its target genes to help cell survival and recovery. Due to rapid growth, proliferation, and hypoxia in tumor microenvironment, cancer cells often exhibit an elevated ISR compared to normal cells, and its upregulation in cancer cells contributes to their survival, growth, and resistance to drug treatments. However, if the cellular stress is severe, either in intensity or in duration, ATF4 regulates the expression of another set of genes to execute cell death (114–116) (Figure 4).

ATF4 is a key effector of cell fate in response to the ISR. When ATF4 is not bound to its DNA target, it exists as a monomer (117). ATF4 can interact with bZIP or AP-1 transcription factors to form heterodimers. Transcriptional selectivity of ATF4 is modulated by the formation of heterodimers with CHOP or ATF3, both of which are transcriptional targets of ATF4. ATF4-ATF3 heterodimers regulate various cellular processes, including stress responses, apoptosis, and autophagy. ATF4-CHOP heterodimers mainly regulate apoptosis (112, 118–120). 

ATF4 regulates CHOP gene expression, which is important for mediating ISR-induced apoptosis. CHOP is a transcription factor belonging to the bZIP family. CHOP induces apoptosis by upregulating BIM, PUMA, NOXA, and DR5, affecting both the intrinsic and extrinsic pathways (114, 115). ATF4 itself can promote apoptosis by directly upregulating NOXA and PUMA expression, leading to cancer cell apoptosis (75, 121, 122). Also, ATF4 promotes XIAP protein degradation through the ubiquitin-proteasome system, ensuring apoptosis together with CHOP upregulation (123). CHOP-ATF3 heterodimers can increase the transcription of DR5, thus promoting apoptosis (124). ATF4-CHOP heterodimers regulate the expression of proapoptotic genes such as *PUMA*, *NOXA*, and *APAF1* (125, 126).

### ONC201

ONC201 is a first-in-class imipridone compound that is currently being tested in clinical trials for various malignancies, including leukemia, lymphoma, and colon, prostate, breast, and CNS tumors (127). The drug was originally discovered as a TRAIL-inducing compound (TIC10) in a chemical library screen and was shown to inhibit cancer cell viability (128). The most well-characterized imipridones include ONC201, ONC206, and ONC212. The clinical trials demonstrate that ONC201 is a well-tolerated and potent anticancer drug in previously treatment-resistant patients (129). ONC201 shows potent inhibitory effects on H3K27M-mutated diffuse midline glioma (DMG) (130–133). The encouraging preliminary clinical activity in DMG led to an ongoing international, randomized phase III trial with ONC201 for the treatment of newly diagnosed H3 K27M–mutant diffuse glioma following completion of radiotherapy (NCT05580562). Another trial is investigating ONC206 in adults with recurrent primary CNS tumors (NCT04732065) (Table 2).

As mentioned above, ONC201 was originally called TRAIL-inducing compound 10 (TIC10) and was later discovered to activate the ISR, causing cell death through upregulation of the TRAIL/DR5 extrinsic pathway and ATF4 (128, 134). Studies have indicated multiple pathways as putative mechanisms, including dopamine receptor antagonism, activation of the TRAIL-mediated extrinsic pathway, and regulation of the ISR. Here, we focus on the ISR-mediated effects of imipridones. In an effort to search for the direct targets of imipridones, ONC201 and ONC212 were found to act as potent activators of caseinolytic mitochondrial matrix peptidase proteolytic subunit (ClpP) (135, 136). ClpP localizes to the mitochondrial matrix and is essential for homeostasis of mitochondrial proteins. ClpX acts as a substrate-recognizing subunit for the ClpP protease, forming the ClpXP complex. This complex is essential for protein degradation (137) (Figure 4).

The crystal structure of the ONC201-ClpP complex indicates that ONC201 binds to the hydrophobic pockets between adjacent ClpP subunits. This binding disrupts the protein-protein interaction between ClpP and ClpX and induces opening of ClpP’s axial entrance pore, which is normally opened by ClpX. ONC201 causes ClpP’s entrance pore radius to enlarge from 12 to 17Å. As a result, ONC201 activates ClpP in the absence of ClpX (135, 136). Activated ClpP cleaves many mitochondrial proteins, including those required for oxidative phosphorylation, resulting in mitochondrial stress, leading to activation of the ISR and ATF4 upregulation (135, 136) (Figure 4). But the mechanism connecting ClpP activation to ATF4 upregulation still is unknown. The ONC201 analog, ONC212, has a highly electronegative p-CF3 benzyl substituent that extends into ClpP’s apolar pocket and enhances affinity with the protease (136). That enhanced affinity is consistent with the observation that ONC212 is about 10-fold more potent than ONC201.

Imipridone treatment induces gene-expression profiles consistent with ISR activation, mainly by upregulating the expression of ATF4 (134) Imipridones trigger typical or atypical ISR, depending on cancer cell type. For example, in colorectal (134), AML (138), and breast cancer cells (139), imipridones induce typical ISR. On the other hand, in MCL (138) and cutaneous T cell lymphoma (CTCL) (140), imipridones activate atypical ISR. The mechanisms of atypical ISR activation also remain elusive.

### Bortezomib and carfilzomib

The proteasome is a large protease complex that degrades many cellular proteins via a ubiquitin-dependent system (141, 142). MM is an incurable clonal B cell malignancy characterized by the accumulation of terminally differentiated, antibody-producing plasma cells in the bone marrow (143). Bortezomib was the first-in-class compound to be approved by the FDA for MM and is a cornerstone of antimyeloma therapy (144, 145). Carfilzomib is a second-generation proteasome inhibitor with an improved efficacy and safety profile compared with bortezomib (146) (Table 2).

Bortezomib is a reversible inhibitor of the proteasome with a peptide-like backbone and boronated group. In contrast, carfilzomib is an irreversible proteasome inhibitor that contains an epoxyketone as an active group (146). Inhibition of the proteasome leads to the accumulation of polyubiquitinated misfolded or unfolded proteins (PUMUP), which leads to ER stress and upregulation of ATF4 through the ISR. Thus, ATF4-mediated apoptosis is an important mechanism of proteasome inhibitors (147, 148) (Figure 4). However, acquired or secondary resistance consistently emerges in patients who initially respond to proteasome inhibitors (149). Two resistance mechanisms have been identified (Figure 4). Inhibition of the proteasome promotes the degradation of unfolded and misfolded proteins through the aggresome pathway, which relieves the accumulation of unwanted proteins and the ISR (147, 150). Polyubiquitinated proteins (PUPs) in association with HDAC6 bind to dynein motor protein. The PUP-HDAC6-dynein complex moves to the aggresome along the microtubule. Aggresome formation ultimately induces autophagic clearance, which terminates in lysosomal degradation (147, 150). Therefore, the dual inhibition of HDAC6 and the proteasome triggers dramatic and prolonged accumulation of unwanted proteins and induces apoptosis in resistant myeloma cells (RPMI-8226v10r, Kas6v10r, RPMI-LR5, and RPMI-Dox40) (147, 151, 152). ER stress induced by proteasome inhibitors can also promote HDAC4 binding to ATF4 to prevent its nuclear translocation, hence inhibiting ATF4 transcriptional activity and leading to cells resistant to bortezomib or carfilzomib treatments (152–154). Dual inhibition of HDAC4 and proteasome synergistically activates ATF4-mediated cell apoptosis (153–155).

### PG3-Oc and CB002 preclinical development

The third approach mentioned above aims at restoring expression of proapoptotic p53 target genes in a p53-independent way in p53-deficient tumors. These approaches may be broadly applicable, as WT p53, p53-deleted, and p53-mutated tumors could all be targeted. Compound PG3-Oc is an analogue of the natural product prodigiosin, and it triggers ISR and leads to activation of ATF4 (Figure 4). ATF4 regulates the expression of a subset of p53 target genes in p53-deficient HCT116^–/–^ and p53-mutated HT29 cells, including *PUMA*, *DR5*, *NOXA*, and *CDKN1A* (encoding p21). Among them, PUMA plays an important role in mediating cancer cell apoptosis (75).

CB002 and its derivatives are xanthine analogs. They induce ISR and ATF4-mediated expression of NOXA and DR5 (Figure 4). NOXA is responsible for cell apoptosis (74). Transcriptomic and proteomic analyses show that PG3-Oc and CB002 upregulate transcriptomes and proteomes that overlap with the p53 target gene database. Importantly, the overlapping gene sets contain typical p53 target genes that regulate cell cycle and apoptosis as mentioned above. Although p53 and ATF4 generally control different genes, they converge on a set of common transcriptional targets related to apoptosis. A recent paper studied shared gene targets of ATF4 and p53 transcriptional networks (156). Authors report that the p53 and ISR pathways converge to independently regulate common metabolic and proapoptotic genes. They demonstrate that these targets require p53 during DNA-damage response, but not during the ISR. In contrast, ATF4 is required during the ISR and is dispensable under p53-activating conditions (156). These results provide a rationale for combined treatments of DNA-damaging drugs or MDM2 inhibitors with ISR inducers to achieve synergistic antitumor effects in WT p53 tumors. Andrysik et al. reported that inhibition of the phosphatase PPM1D led to activation of ATF4 through ISR (157). Nelfinavir is an inhibitor of HIV-1 protease and a robust ISR inducer (158). PPM1D inhibitor or nelfinavir synergized with MDM2 inhibitors to amplify expression of some p53 targets and synergistically increase cell death in vitro and in HCT116 tumor xenografts (157).

## Conclusions

Dysregulation of and resistance to apoptosis is a hallmark of cancer cells due to mutations in the extrinsic, intrinsic, p53, and ISR pathways. Targeting these apoptotic pathways is an intriguing approach to identifying new antitumor therapies. The ability to target and activate apoptosis in resistant tumor cells will continue to evolve in future clinical practice. The future development of agents that target apoptotic pathways either directly or indirectly through the p53 and ISR pathways could lead to disease regression or cures in patients with difficult-to-treat tumors.

## Figures and Tables

**Table 2:**
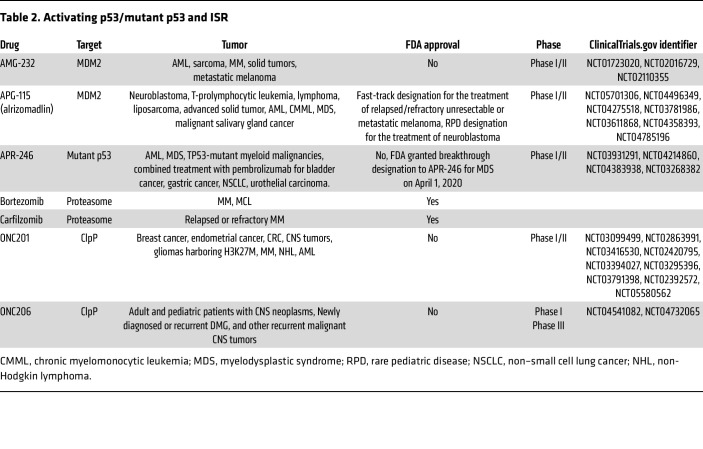
Activating p53/mutant p53 and ISR

**Table 1:**
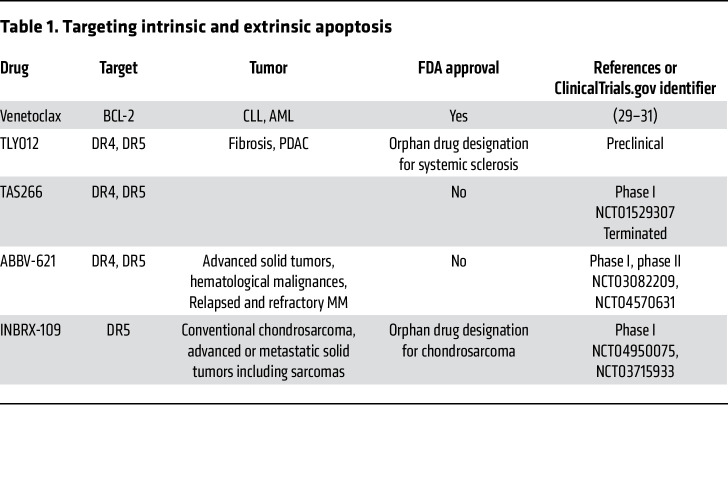
Targeting intrinsic and extrinsic apoptosis

## Version 1 07/15/2024

Electronic publication

## Version 2 07/15/2025

Correction with corrigendum
