# Corrigendum to Targeting apoptotic pathways for cancer therapy

**DOI:** 10.1172/JCI196275

**Published:** 2025-07-15

**Authors:** Xiaobing Tian, Praveen R. Srinivasan, Vida Tajiknia, Ashley F. Sanchez Sevilla Uruchurtu, Attila A. Seyhan, Benedito A. Carneiro, Arielle De La Cruz, Maximilian Pinho-Schwermann, Andrew George, Shuai Zhao, Jillian Strandberg, Francesca Di Cristofano, Shengliang Zhang, Lanlan Zhou, Alexander G. Raufi, Arunasalam Navaraj, Yiqun Zhang, Nataliia Verovkina, Maryam Ghandali, Dinara Ryspayeva, Wafik S. El-Deiry

Original citation: *J Clin Invest*. 2024;134(14):e179570. https://doi.org/10.1172/JCI179570

Citation for this corrigendum: *J Clin Invest*. 2025;135(14):e196275. https://doi.org/10.1172/JCI196275

Following publication of this Review, the Editors became aware that portions of the text overlapped with text in prior publications by others (1–7) and by some of the Review authors (8, 9). To meet the originality requirements of the *JCI*, the authors have rewritten those portions of the review. The HTML and PDF versions have been updated online. In addition, the Journal has published an online version of the original article marking the edited text (Supplemental File, Redaction).

The authors regret the errors.

## Supplementary Material

Supplemental data

## Version 1 07/15/2025

Electronic publication
