# Supplementary material for: Corrigendum to Targeting apoptotic pathways for cancer therapy
Source: J Clin Invest. 2025 Jul 15;135(14):e196275. doi: 10.1172/JCI196275 (PMC12259242; doi:10.1172/JCI196275)
Supplement: Supplemental data [file jci-135-196275-s217.pdf]

Targeting apoptotic pathways for cancer therapy

Xiaobing Tian,[1,2,3,4] Praveen R. Srinivasan,[1,2,3,4] Vida Tajiknia,[1,2,3,4] Ashley F. Sanchez Sevilla Uruchurtu,[1,2,3,4,5] Attila A. Seyhan,[1,2,3,4] Benedito A. Carneiro,[1,3,4,6] Arielle De La Cruz,[1,2,3,4] Maximilian Pinho-Schwermann,[1,3,4,6] Andrew George,[1,2,3,4] Shuai Zhao,[1,2,3,4] Jillian Strandberg,[1,2,3,4] Francesca Di Cristofano,[1,2,3,4] Shengliang Zhang,[1,2,3,4] Lanlan Zhou,[1,2,3,4] Alexander G. Raufi,[1,3,4,6], Arunasalam Navaraj,[1,2,3,4] Yiqun Zhang,[1,2,3,4] Nataliia Verovkina,[1,2,3,4] Maryam Ghandali,[1,2,3,4] Dinara Ryspayeva,[1,2,3,4] and Wafik S. El-Deiry[1,2,3,4,5,6]

[1]Laboratory of Translational Oncology and Experimental Cancer Therapeutics and [2]Department of Pathology and Laboratory Medicine, Warren Alpert Medical School, Brown University, Providence, Rhode Island, USA. [3]Joint Program in Cancer Biology, Lifespan Health System and Brown University, Providence, Rhode Island, USA. [4]Legorreta Cancer Center at Brown University, Providence, Rhode Island, USA. [5]Pathobiology Graduate Program, Brown University, Providence, Rhode Island, USA. [6]Hematology/Oncology Division, Department of Medicine, Lifespan Health System and Brown University, Providence, Rhode Island, USA.

Apoptosis is a form of programmed cell death that is mediated by intrinsic and extrinsic pathways. Dysregulation of and resistance to cell death are hallmarks of cancer. For over three decades, the development of therapies to promote treatment of cancer by inducing various cell death modalities, including apoptosis, has been a main goal of clinical oncology. Apoptosis pathways also interact with other signaling mechanisms, such as the p53 signaling pathway and the integrated stress response (ISR) pathway. In addition to agents directly targeting the intrinsic and extrinsic pathway components, anticancer drugs that target the p53 and ISR signaling pathways are actively being developed. In this Review, we discuss selected and promising anticancer therapies in various stages of development, including drug targets, mechanisms, and resistance to related treatments, focusing especially on B cell lymphoma 2 (BCL-2) inhibitors, TRAIL analogues, DR5 antibodies, and strategies that target p53, mutant p53, and the ISR.

Introduction

Apoptosis is a form of regulated cell death with a critical role in development and homeostasis (1). Activation of apoptotic pathways results in destruction of target cells with minimal inflammatory response and disruption to surrounding tissue. Preventing cancer is an important function of apoptosis (2), and dysregulation and evasion of apoptosis are hallmarks of cancer. Tumor cells employ multiple mechanisms to evade apoptosis, including expression of apoptosis inhibitors as a means of acquiring resistance to cancer therapies. Much effort has gone into developing drugs to reinstate or promote apoptosis in cancer cells. Below, we will briefly describe the major apoptotic pathways, then highlight major advancements toward targeting these pathways and other regulators of apoptosis in cancer cells.

Intrinsic and extrinsic apoptotic pathways

Two pathways are considered the major drivers of apoptosis in all cells: the intrinsic pathway, initiated by the formation of Bax and Bak pores on the mitochondrial outer membrane (MOM), and the extrinsic pathway, triggered by death receptors (DRs) on the plasma membrane (Figure 1).

Conflict of interest: WSED is the scientific founder and shareholder of Oncoceutics Inc. (acquired by Chimerix), p53-Therapeutics Inc., and SMURF-Therapeutics Inc. BAC receives institutional research support from AstraZeneca, Abbvie, Actuate Therapeutics, Astellas, Agenus, Bayer, Dragonfly Therapeutics, Mink Therapeutics, Pfizer, Pyxis Oncology, Repare Therapeutics, and Regeneron.

Copyright: © 2024, Tian et al. This is an open access article published under the terms of the Creative Commons Attribution 4.0 International License.

Reference information: *J Clin Invest*. 2024;134(14):e179570.
https://doi.org/10.1172/JCI179570.

Intrinsic apoptosis

In most mammalian cells, the B cell lymphoma 2 (BCL-2) protein family regulates the intrinsic pathway (Figure 1A) (3). BCL-2 family members are characterized by the presence of up to four distinct segments of amino acid homology, termed BCL-2 homology (BH) domains. The interactions of the BCL-2 protein family are depicted in detail in Figure 2A (3–8).

Extrinsic apoptosis

Perturbations of the extracellular microenvironment that trigger release of Fas-L, TNF, and TRAIL activate the extrinsic apoptotic pathway when these extracellular ligands bind to Fas, TNF receptors, and DR4/5, respectively. As ongoing efforts in anticancer drug discovery and development continue to focus on targeting DR4/5, we will focus on their role in apoptosis here. The mechanism of DR4/5 activation is summarized in Figure 1B (9–14).

IAPs and execution of apoptosis

Inhibitors of apoptosis (IAPs) constitute a highly conserved family of proteins defined by the presence of 1–3 protein motifs called baculovirus IAP repeats (BIRs). Most BIRs form a surface hydrophobic groove that specifically binds a conserved tetrapeptide motif, called IAP-binding motif (IBM), found in the active subunits of caspase-3, -7, and -9 (15). Second mitochondrial activator of caspase (SMAC) released by MOM permeabilization blocks IAPs (including XIAP) to promote cell death (16) (Figure 1A). Caspases-3, -6, and -7 execute apoptosis via the proteolysis of thousands of cellular proteins. The main features of cells undergoing apoptosis include chromatin condensation, DNA fragmentation, membrane blebbing, and cytoskeletal rearrangement (4).

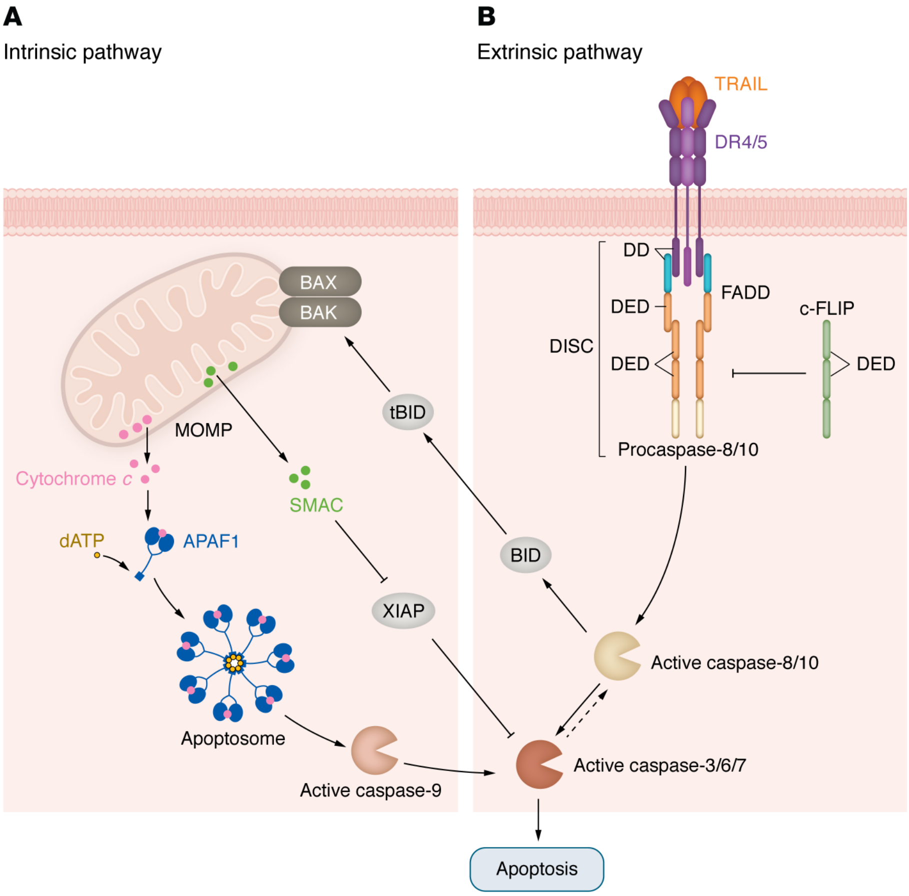

Figure 1. Intrinsic and extrinsic apoptosis pathways. (**A**) Intrinsic apoptosis pathways. Upon activation, BAK and BAX undergo conformational changes and oligomerization, forming pores in the MOM and causing irreversible MOM permeabilization (MOMP), the critical step for intrinsic apoptosis (3), allowing release of cytochrome *c* and SMAC. Cytochrome *c* and dATP join APAF-1 and the initiator protein procaspase-9 to form the apoptosome, while SMAC interacts with IAPs (see below). Within the apoptosome, procaspase-9 is cleaved into active caspase-9, which cleaves and activates the apoptosis effector proteins caspase-3, -6, and -7 (3). (**B**) Extrinsic apoptosis pathway. Upon ligand binding, DR4 and DR5 trimerize and aggregate within the cell membrane, a process known as capping. This is followed by recruitment of the adaptor protein FADD, which has a death effector domain (DED). Initiator procaspase-8 and -10 also have DEDs that bind to FADD at its DED, forming the DISC. Procaspase-8 and -10 are activated within the DISC and in turn cleave and activate executioner caspase-3, -6, and -7. Activation of procaspase-8/10 is negatively regulated by c-FLIP. c-FLIP competes directly with procaspase-8 for binding to FADD through homotypic DED interactions, thus inhibiting procaspase-8 recruitment and activation at the DISC (9-12). Activated caspase-8 also cleaves the BH3 subfamily member BID to active form truncated-BID (tBID). tBID translocates to the MOM and initiates apoptosis through its interactions with proapoptotic effector proteins BAK and BAX. BID cleavage and translocation to the mitochondria link the extrinsic cell death pathway to the intrinsic apoptotic pathway and amplify the apoptotic response. This amplification mechanism is required for effective apoptosis in certain cells, denoted as type II cells for their mechanism of apoptosis, in contrast with type I cells, which undergo extrinsic apoptosis independently of intrinsic apoptosis pathway induction (13, 14).

Targeting intrinsic apoptosis in cancer therapy

Cancer cells resist apoptosis using a variety of mechanisms. Defects in intrinsic pathways include the following: (a) acquiring of caspase gene mutations that inhibit caspase function (2); (b) overexpression of antiapoptotic BCL-2 family proteins (2, 15); (c) overexpression of IAPs (17); (d) loss and inactivation of apoptotic effectors BAX and BAK (2, 18); (e) insufficient release of cytochrome *c* and mutation of lysine residues (especially K72) of cytochrome *c* that abrogate the apoptosome formation, causing inhibition of caspase activation (19, 20); and (f) defects in extrinsic pathway signaling. These defects include (a) overexpression of apoptosis-inhibiting decoy receptors (e.g., DcR1/2), which compete with DR4/5 for TRAIL binding (21); (b) decreased DR4/5 activity; and (c) death-inducing signaling complex (DISC) inhibition by FLICE-like inhibitory protein (c-FLIP) (22). For instance, colorectal cancer (CRC) cells have decreased activity of DR4/5 that contributes to their resistance to apoptosis (21, 23). Decreased expression of DR4/5 seems to result from defective p53, impaired transport from ribosomes, defective redistribution of DR4/5 in lipid rafts and mutations, epigenetic changes (23, 24), or overexpression of DcR3.

Tumor cells can overexpress multiple inhibitors of both apoptotic pathways, including in the process of acquiring resistance to cancer therapy. Upregulation of the antiapoptotic BCL-2 family proteins and decreased expression of proapoptotic proteins are responsible for cancer cell resistance to chemotherapy and radiation. For example, BCL-2 gene expression is elevated in over half of all cancers and XIAP is overexpressed in many tumors (2, 4, 17).

Recent development of apoptosis-targeted drugs has focused on the intrinsic pathway, including BCL-2, MCl-1, and IAP inhibitors (25). In this Review, we focus our discussion on BCL-2–specific inhibitors due to the relatively recent approval of the BCL-2 inhibitor venetoclax by the US FDA.

Venetoclax

BCL-2 inhibitors, also known as BH3 mimetics, are among the frontrunners of agents that were developed as a targeted approach to directly alter the intrinsic apoptosis pathway. BH3 mimetics are small molecules that mimic the binding of BH3-only proteins to the hydrophobic pockets within antiapoptotic BCL-2 proteins. In 2016, venetoclax (ABT-199) was the first agent targeting BCL-2 to be approved by the US FDA for the treatment of patients with chronic lymphocytic leukemia (CLL) harboring 17p deletion. Venetoclax binds to BCL-2, leading to the release of BIM, which in turn directly activates BAX and BAK (26–28) (Table 1 and Figure 2A). In May 2019, venetoclax was approved by the FDA for the frontline treatment of patients with CLL owing to the superior efficacy of venetoclax plus the anti-CD20 antibody obinutuzumab over chlorambucil plus obinutuzumab, thus providing a chemotherapy-free option for CLL

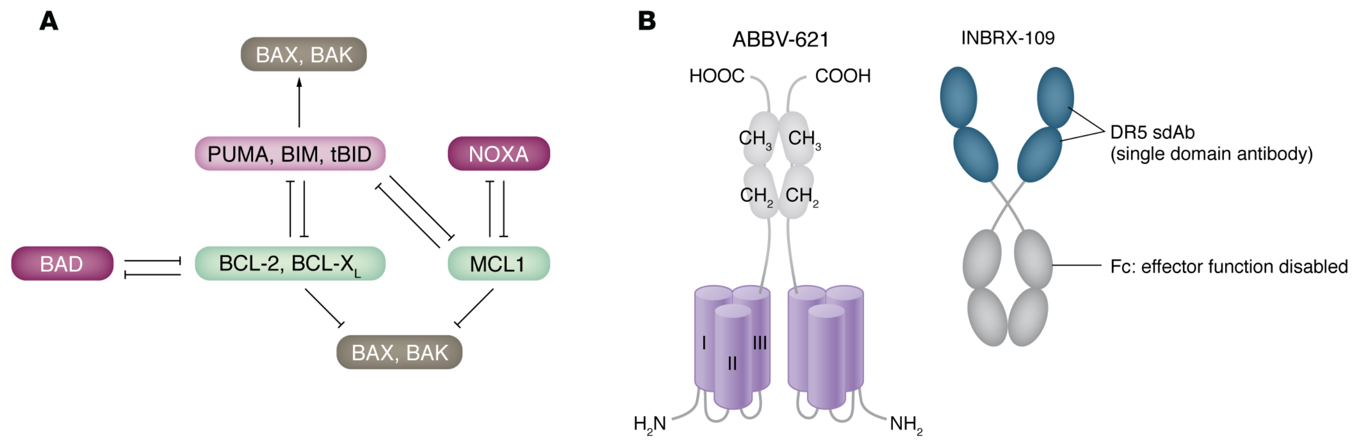

Figure 2. Targets in the intrinsic and extrinsic apoptosis pathways. (**A**) Interactions of the BCL-2 protein family. The multi-BH domain family members either suppress apoptosis (e.g., BCL-2, BCL-XL, and MCL-1) or promote apoptosis (e.g., BAX, BAK), whereas the BH3-only subfamily members identified to date (e.g., BAD, BID, PUMA, NOXA, and BIM) function exclusively to promote cell death (3, 4) BH3-only proteins can be divided into activators or sensitizers. The activators PUMA, tBID, and BIM directly activate BAK and BAX and interact with antiapoptotic proteins to promote MOMP (5, 6). In contrast, the sensitizers BAD and NOXA only interact with the antiapoptotic proteins and do not activate BAK and BAX (7, 8). Interactions with antiapoptotic BCL-2 proteins and activator BH3-only proteins regulate BAK and BAX activity. (**B**) High-potency TRAIL receptor agonists. ABBV-621 is a hexavalent TRAIL fusion protein with Fc-FcγR interactions disabled by IgG Fc D297S mutation. INBRX-109 is a tetravalent DR5 agonistic antibody with Fc effector function disabled by forming a cycle.

patients (29). Venetoclax was also approved by the FDA in 2020 for the treatment of elderly patients with newly diagnosed acute myeloid leukemia (AML) who are ineligible for standard induction chemotherapy (i.e., >75 years old) (30, 31).

Targeting extrinsic apoptosis in cancer therapy

TRAIL analogs

TRAIL is a transmembrane trimeric glycoprotein that can be cleaved by metalloproteinases and released as a soluble factor (32, 33). Both soluble and membrane-bound forms of TRAIL can bind to DR4/5, triggering the extrinsic apoptosis pathway (Figure 1A). TRAIL signaling selectively induces cancer cell apoptosis without causing toxicity to normal cells. Based on this unique activity profile, many agents targeting this pathway, including recombinant human TRAIL (rhTRAIL, or dulanermin) and DR4/5 agonist antibodies (lexatumumab and conatumumab for DR5, mapatumumab for DR4), have been developed and evaluated in vitro and in vivo (34–37). Preclinical data indicated that both classes of molecules are generally well tolerated, but ultimately, they showed limited anticancer activity in patients. One factor contributing to limited anticancer activity is rhTRAIL's very short half-life in blood, from 0.56 to 1.02 hours (38, 39). Although rhTRAIL induces trimerization (also known as lower-order trimerization) of DR4/5, its soluble form of TRAIL has limited capacity to induce high-order clustering of the DR trimers, resulting in a weak apoptotic signal (40). For DR4/5 agonist antibodies, lower-order receptor trimerization is a major limitation due to the bivalent structure of the antibodies (40, 41).

TLY012. Second-generation rhTRAIL therapeutics were developed to address the clinical limitations of previous TRAIL or TRAIL receptor agonist antibodies. One such conjugate is TLY012, where attaching polyethylene glycol (PEG) to the N-terminus of rhTRAIL increases its size, thereby reducing its clearance by renal filtration (Table 1). This modification prolongs the half-life of TLY012 to 12 to 18 hours, resulting in greater antitumor effect both in vitro and in vivo in CRC models (42). TLY012 also had marked activity against fibrotic cells, characterized by increased expression of DR5 (43). These results supported the orphan drug designation by the FDA in 2019 for the treatment of systemic sclerosis.

Pancreatic cancer cells are notoriously resistant to extrinsic TRAIL-induced apoptosis and undergo type II extrinsic apoptosis (44, 45). TRAIL resistance in pancreatic cancer cells occurs partially due to their overexpression of various IAP family proteins (e.g., cIAP-1, XIAP, and survivin) that block the cleavage of caspase-3, -7, or -9 (46) (Figure 1A). cFLIP blocks TRAIL-induced caspase-8 activation by competing with caspase-8 for binding to Fas-associated death domain (FADD) (25). To this end, ONC201 is a TRAIL- and DR5-inducing compound that may help overcome resistance to TRAIL-induced apoptosis. The combination of ONC201 and TLY012 can induce selective, synergistic apoptosis in six pancreatic cancer cell lines and significantly delays tumor xenograft growth in vivo (47). The combination of TLY012 and PD-1 immune checkpoint inhibition also reduced the growth of pancreatic tumors in vivo and promoted tumor infiltration of $CD8^+$ T cells, suggesting the potential of TLY012 to enhance the effects of checkpoint inhibitors (48).

Eftozanermin alfa (ABBV-621). In clinical studies of TRAIL derivatives and DR4/5 agonists, overall response rates did not meet the requirements to pass clinical trials (36, 38, 39, 49–54). DR4/5 agonist antibodies show potent antitumor effects in various tumor xenograft models, but not in clinical trials. There are two reasons: (i) rhTRAIL, TRAIL derivatives, and DR4/5 agonist antibodies do not induce high-order receptor clustering, which reduces apoptosis signaling; and (ii) IgG can cross-link Fc region of the antibodies to Fcγ receptors (FcγRs). Mouse models have lower IgG levels than do cancer patients. ~~although antitumor activity was observed for individual patients, overall response rates were disappointing and could not confirm the promising preclinical~~

Table 1. Targeting intrinsic and extrinsic apoptosis

Drug	Target	Tumor	FDA approval	References or ClinicalTrials.gov identifier
Venetoclax	BCL-2	CLL, AML	Yes	(29–31)
TLY012	DR4, DR5	Fibrosis, PDAC	Orphan drug designation for systemic sclerosis	Preclinical
TAS266	DR4, DR5		No	Phase I NCT01529307 Terminated
ABBV-621	DR4, DR5	Advanced solid tumors, hematological malignances, Relapsed and refractory MM	No	Phase I, phase II NCT03082209, NCT04570631
INBRX-109	DR5	Conventional chondrosarcoma, advanced or metastatic solid tumors including sarcomas	Orphan drug designation for chondrosarcoma	Phase I NCT04950075, NCT03715933

~~results (36, 38, 39, 49–54). Despite the potent antitumor efficacy of all DR4/5 agonists in xenograft tumor models derived from various human cancer cell lines during preclinical development, translation into the clinical setting has not yet been successful.~~

~~A major limitation of both first- and second-generation rhTRAIL, receptor-specific mAbs, and TRAIL derivatives is their inability to induce efficient lower- and higher-order receptor clustering, leading to reduced apoptotic signaling. It has been shown that due to the bivalent structure of agonistic antibodies, additional crosslinking of the Fc region of the antibodies to Fcγ receptors (FcγR) is necessary for high clustering capacity and efficient antitumor response in vivo in xenograft models (55, 56). However, IgG is known to compete with these agonistic antibodies for FcγR. Mouse models have very low levels of IgG compared with levels in cancer patients.~~ In patients, high concentrations of endogenous IgG compete for FcγR binding, inhibiting efficient clustering of agonistic antibodies (55, 56).

To address this problem, APG350 was engineered to potently increase receptor clustering for full antitumor activity independently of FcγR. It contains two single-chain TRAIL receptor–binding domains (scTRAIL-RBD), and each scTRAIL-RBD carries three binding sites for a receptor, resulting in a dimer with six binding sites for DR4/5 (Figure 2B). This structural change improves low-order clustering ability and leads to induction of efficient high-order clustering ~~APG350 was shown to have an enhanced lower-order clustering efficiency as compared with DR4/5 mAbs, and because it can simultaneously bind two DR trimers, it has a greater ability to induce higher-order receptor clustering as compared with rhTRAIL and its derivatives~~ (55, 57).

~~Eftozanermin alfa is a derivative of APG350 engineered as an IgG1-Fc mutant backbone linked to two sets of trimeric native single-chain TRAIL receptor–binding domain monomers (Figure 2B). It selectively binds to TRAIL receptors with nanomolar affinity to induce optimal receptor clustering in human solid tumor cancer cells, driving on-target apoptosis and robust antitumor activity independently of Fc–FcγR interactions. Eftozanermin alfa was well tolerated in patients with advanced solid tumors and hematological malignancies when administered alone and in combination with venetoclax or chemotherapeutics~~ Modification of APG350 yielded eftozanermin alfa by introducing a mutation into the IgG1-Fc binding site and eliminating Fc-FcγR interaction (Figure 2B). Eftozanermin alfa shows highly specific and strong binding to death receptors and induces optimal receptor clustering, leading to on-target apoptosis in vitro and in vivo. Also, clinical trials show that eftozanermin alfa has fewer side effects and is well tolerated in patients with various cancers (57, 58).

In the 105 patients with advanced solid tumors who were studied, eftozanermin alfa monotherapy led to tumor responses in two patients with CRC and one with pancreatic cancer (58). The combination of eftozanermin alfa and venetoclax was investigated in patients with refractory AML and showed an encouraging response rate of 30%, including four complete responses (58). Eftozanermin alfa fully occupies the binding sites on the death receptors, leading to enhanced cleavage of PARP and apoptosis. Importantly, the combination treatment also induced more tumor infiltration of immune cells, such as $CD4^+$ T cells, in treated tumor specimens. ~~Pharmacodynamic studies demonstrated saturation of eftozanermin alfa–binding sites on the TRAIL receptors and increased levels of M30 and M65 markers of apoptosis in serum. Analysis of paired tumor specimens collected during the clinical trial showed increased tumor infiltration of immune cells including~~ ~~$CD4^+$ T cells in posttreatment biopsies compared with baseline tumor specimens as well as increased PARP cleavage and downregulation of the MEK/ERK1/2/AKT pathway.~~ Despite these encouraging results demonstrating target engagement and signal of clinical activity, the only active clinical trial with eftozanermin listed at ClincalTrials.gov is a phase II trial investigating eftozanermin alfa plus bortezomib and dexamethasone for patients with multiple myeloma (MM) (NCT04570631) (Table 1).

Agonistic DR5 antibodies

TAS266. ~~Nanobodies are a novel class of therapeutic proteins based on high-affinity single variable domains (VHH) derived from heavy chain antibodies occurring naturally in camelids that can be linked to form multivalent molecules~~ Nanobodies are small, single-domain antibody fragments derived from camelids that have a high affinity for their target antigen (59). TAS266 is an agonistic tetravalent nanobody targeting DR5 consisting of four identical humanized VHH antibody fragments connected through three linkers. TAS266 specifically binds to and activates DR5 receptors, leading to apoptosis. It is more potent than cross-linked DR5 antibodies or TRAIL for inducing cell apoptosis ~~Each VHH monomer domain of TAS266 can bind with high affinity to a DR5 molecule. TAS266 can cluster four DR5 molecules or bridge two DR5 trimers, initiating more rapid DISC formation and downstream apoptotic signaling as compared with other conventional DR5 agonists or TRAIL~~ (41). In vivo, TAS266 elicited single-dose

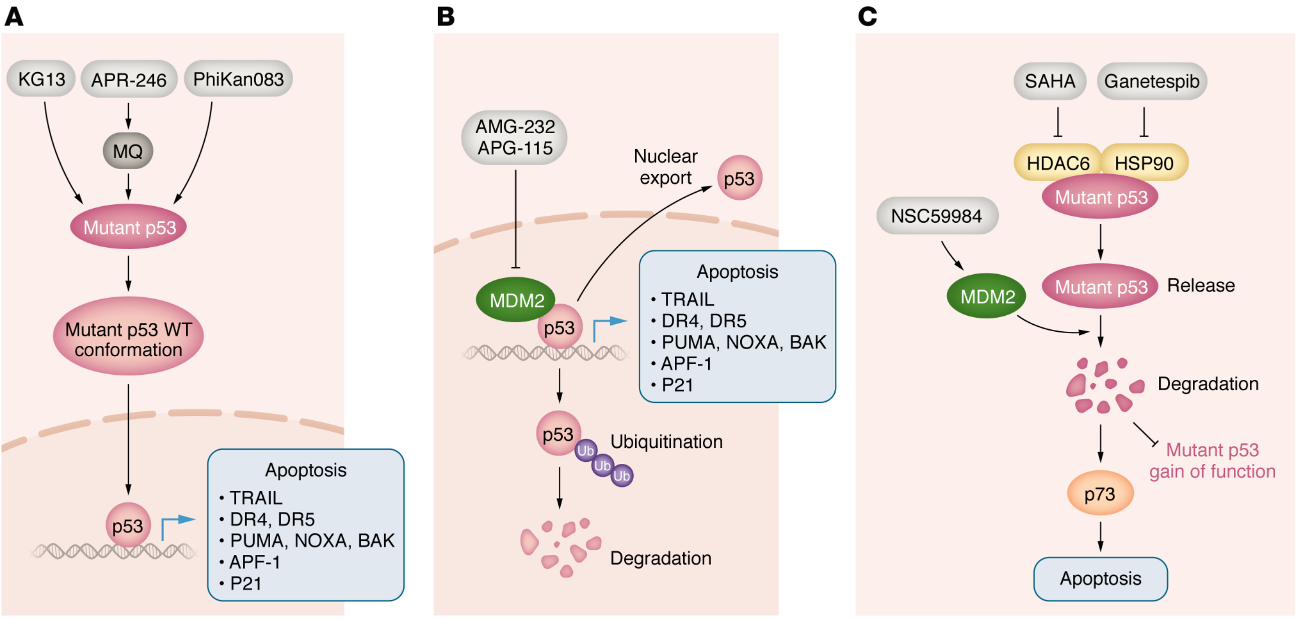

Figure 3. Strategies targeting p53 and mutant p53. (**A**) Reactivation of mutant p53. Direct binding of a small molecule (gray boxes) to a mutant p53 promotes and stabilizes WT p53 folding and conformation, leading to restoration of specific DNA binding and transcription of p53 target genes. This will induce tumor cell apoptosis or senescence. (**B**) Inhibition of MDM2. MDM2 binds to p53 directly through its N-termini and inhibits p53 function through two major mechanisms: (a) upon binding, MDM2 ubiquitinates p53, promoting proteasomal degradation of p53; (b) MDM2 promotes export of p53 out of the cell nucleus. (**C**) Depletion of mutant p53. Small molecules inhibit MTp53 gain-of-function and dominant-negative effects through degradation of MTp53.

tumor regressions in multiple human tumor xenograft models (59). However, in a phase I clinical trial, TAS266 showed severe hepatotoxicity that was attributed to hyperclustering by preexisting antidrug antibodies (ADAs), leading to suspension of the clinical trial and development of this drug (41).

INBRX-109. INBRX-109 is a third-generation, tetravalent agonistic antibody engineered to reduce the hepatotoxicity based on a single domain antibody platform (Figure 2B). It consists of two identical camelid heavy chain–only antibody-binding domains targeting DR5. These domains are joined end to end with an effector-silenced Fc constant domain based on human immunoglobulin G1. INBRX-109's design eliminates recognition by preexisting ADAs (41). In a phase I study, INBRX-109 showed antitumor activity in patients with chondrosarcoma, a rare bone cancer, resulting in a disease control rate of 87% among 31 patients. Two patients had tumor partial responses, a rare positive outcome with this tumor type, which is resistant to chemotherapy and radiation therapy, and 25 patients had stable disease (60). The treatment was well tolerated, with low grade liver-related adverse events. These results led to an ongoing randomized phase II trial of INBRX-109 in conventional chondrosarcoma (NCT04950075). In 2021, the FDA granted fast-track designation to INBRX-109 for the treatment of patients with unresectable or metastatic chondrosarcoma (Table 1).

Targeting p53 and mutant p53 in cancer therapy

p53 is the guardian of the genome and an important upstream regulator of apoptosis and other key biological functions (61). The essential growth-arrest and proapoptotic genes induced by activated p53 include *CDKN1A* (p21), *PUMA*, *NOXA*, *BAK*, apoptotic protease-activating factor-1 (*APAF-1*), *TRAIL*, and *DR5* (62–66) (Figure 3). Therefore, p53 affects both intrinsic and extrinsic apoptosis pathways. p53 is inactivated in around 50% of human cancers and in almost all tumor cell lines in culture (67). Two important mechanisms responsible for inactivation of p53 include mutation of the *TP53* gene and negative regulation of WT p53 protein by MDM2. DNA-damaging drugs can potently activate WT p53; however, secondary malignancies due to increased mutation burden remain a substantial concern (68). Restoration of the p53-regulated transcriptome without DNA damage represents an important anticancer strategy. Approaches using this strategy can be divided into three categories. The first approach uses agents targeting p53-negative regulators to activate WT p53, such as MDM2 inhibitors (69, 70). The second approach involves directly targeting mutant p53 by small molecules to restore its conformation and WT p53 function (71–73). The third approach is indirect and bypasses p53 by compounds that upregulate proapoptotic p53 targets in p53-deficient tumors via inducing the integrated stress response (ISR) (74, 75) or activating p73 (76).

Reactivation of suppressed WT p53

MDM2 inhibitors. MDM2 is a nuclear-localized E3 ligase, and its overexpression is common in various cancers. MDM2 binds to and ubiquitinates p53, causing p53 proteasomal degradation and promoting export of p53 out of the cell nucleus (77). In addition, *MDM2* is a p53 target gene and inhibits p53 activity through a feedback mechanism (78) (Figure 3). MDM2 inhibitors bind to the p53-binding pocket in MDM2 and inhibit p53/MDM2 interaction, leading to stabilization of p53 and induction of p53-dependent cell-cycle arrest or apoptosis. The first MDM2 inhibitors identified were nutlins, including nutlin-3a and idasanutlin. Idasanutlin clinical trials were terminated due to futility (NCT03287245 and NCT02545283). Later, other classes of

Table 2. Activating p53/mutant p53 and ISR

Drug	Target	Tumor	FDA approval	Phase	ClinicalTrials.gov identifier
AMG-232	MDM2	AML, sarcoma, MM, solid tumors, metastatic melanoma	No	Phase I/II	NCT01723020, NCT02016729, NCT02110355
APG-115 (alrizomadlin)	MDM2	Neuroblastoma, T-prolymphocytic leukemia, lymphoma, liposarcoma, advanced solid tumor, AML, CMML, MDS, malignant salivary gland cancer	Fast-track designation for the treatment of relapsed/refractory unresectable or metastatic melanoma, RPD designation for the treatment of neuroblastoma	Phase I/II	NCT05701306, NCT04496349, NCT04275518, NCT03781986, NCT03611868, NCT04358393, NCT04785196
APR-246	Mutant p53	AML, MDS, TP53-mutant myeloid malignancies, combined treatment with pembrolizumab for bladder cancer, gastric cancer, NSCLC, urothelial carcinoma. FDA granted breakthrough designation to APR-246 for MDS on April 1, 2020	No	Phase I/II	NCT03931291, NCT04214860, NCT04383938, NCT03268382
Bortezomib	Proteasome	MM, MCL	Yes		
Carfilzomib	Proteasome	Relapsed or refractory MM	Yes		
ONC201	ClpP	Breast cancer, endometrial cancer, CRC, CNS tumors, gliomas harboring H3K27M, MM, NHL, AML	No	Phase I/II	NCT03099499, NCT02863991, NCT03416530, NCT02420795, NCT03394027, NCT03295396, NCT03791398, NCT02392572, NCT05580562
ONC206	ClpP	Adult and pediatric patients with CNS neoplasms, Newly diagnosed or recurrent DMG, and other recurrent malignant CNS tumors	No	Phase I Phase III	NCT04541082, NCT04732065

CMML, chronic myelomonocytic leukemia; MDS, myelodysplastic syndrome; RPD, rare pediatric disease; NSCLC, non–small cell lung cancer; NHL, non-Hodgkin lymphoma.

MDM2 inhibitors were developed (79), such as AMG-232 (80), siremadlin (81), and alrizomadlin (APG-115) (82) (Figure 3 and Table 2). In AML models, APG-115 treatment leads to a reduction in tumor burden and an improvement in survival period. Azacitidine, decitabine, and cytarabine can cause DNA damage and activate p53. Combining APG-115 with these standard treatment drugs demonstrated synergistic effects, which are possibly due to enhanced activation of the p53/p21 pathway ~~APG-115 exerted substantial antileukemic activity, as either a single agent or when combined with standard-of-care (SOC) treatments azacitidine (AZA) and decitabine (DAC) or the DNA-damaging agent cytarabine (Ara-C). By activating the P53/P21 pathway, APG-115 exhibited potent antiproliferative activities and induced cell-cycle arrest in *TP53* WT AML cell lines. In vivo, APG-115 significantly reduced tumor burden and prolonged survival in AML models. Combinations of APG-115 with SOC treatments elicited synergistic antileukemic activity (83). Possibly, APG-115 and SOC agents augment AML cell killing by activating the P53/P21 pathway and upregulating DNA damage~~ (83). A phase 2 clinical trial has been launched to evaluate APG-115 in combination with PD-1 antibody pembrolizumab in patients with solid tumors, including those with *TP53*-mutant tumors (82) (NCT03611868). The clinical trial showed that side effects did not overlap between the 2 drugs, the combination was well tolerated in patients, and APG-115 sensitized the antitumor activity of pembrolizumab. ~~The combination of APG-115 and pembrolizumab was well tolerated in patients with unresectable or metastatic melanoma or advanced solid tumors that have been resistant to immuno-oncologic drugs; adverse events did not overlap between the two agents, according to preliminary results of a phase 2 study.~~ In September 2021, the FDA granted fast-track designation to APG-115 for the treatment of patients with unresectable or metastatic melanoma that is either relapsed or refractory to previous immunotherapy agents. Clinical trials testing the efficacy of MDM2 inhibitors and combination treatments are still ongoing, and the results are yet to be seen.

Although MDM2 is best known for its role in p53 inactivation, this protein also shows p53-independent functions. These include ubiquitination of other proteins (including androgen receptor and transcriptional factor HBP1), regulation of transcription, participation in DNA repair, and regulation of mitochondrial respiration (78, 84–86).

Restoration of mutant p53 function

Eprenetapopt (APR-246). While "boosting WT p53" is a good strategy, it is not applicable to p53-mutated and p53-deleted tumors. Approximately 50%–60% of human cancers carry mutations in the p53 gene (87). The mutations can lead to a loss of normal p53 tumor-suppressor functions and acquisition of new, cancer-promoting functions. These mutant p53 proteins can promote cancer cell proliferation, migration, and invasion, as well as contributing to genetic instability and drug resistance (87, 88). Restoring the p53 signaling pathway involves various strategies ~~Over 50% of human cancers harbor cancer-promoting mutations in p53~~ (87). ~~p53 mutations not only abrogate its tumor-suppressor function, but also confer gain-of-function properties that contribute to tumorigenesis, proliferation, genomic instability, metabolic remodeling, invasion, metastasis, resistance to apoptosis, and cancer-therapy resistance (87, 88). Restoration of the p53 signaling pathway~~

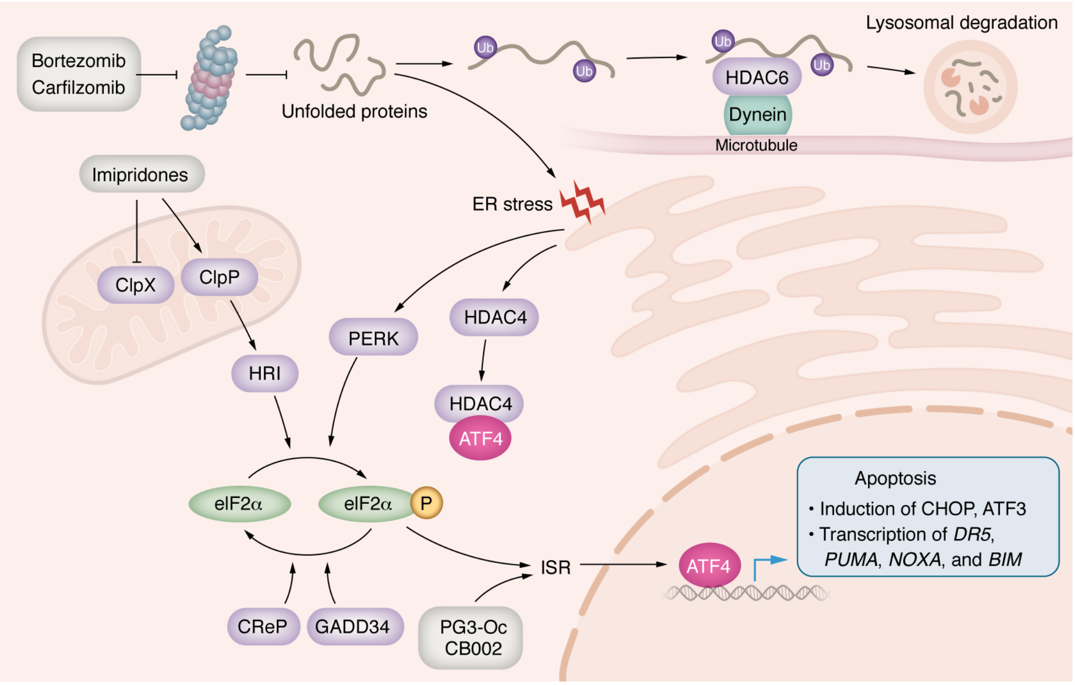

Figure 4. Targeting the ISR and overcoming resistance mechanisms. From top left: in the cell death pathway of the ISR, ATF4 induction can be achieved by eIF2α kinase activators, such as bortezomib, carfilzomib, and imipridones (gray boxes). ATF4 directly or indirectly through the induction of transcriptional factors CHOH or ATF3 regulates the expression of proapoptotic genes, such as DR5, PUMA, NOXA and BIM, which promotes cell apoptosis (lower right). Resistance mechanisms include movement of the PUP-HDAC6-dynein complex to aggresome along the microtubule (upper right). The aggresome is ultimately degraded in lysosomes. Additionally, ER stress induced by the proteasome inhibitors can also promote HDAC4 binding to ATF4 to prevent its nuclear translocation and inhibit ATF4 transcriptional activity.

~~represents an important strategy for achieving successful anticancer therapy in mutant p53–bearing tumors.~~ Eprenetapopt binds to a mutant p53 and induces a conformational change of a mutant p53 protein, leading to WT-p53 conformation and restoring WT function to mutant p53 (87–89).

Eprenetapopt was widely investigated and advanced all the way to phase III trials (89–91). Once eprenetapopt enters cells, it is converted to the reactive electrophile methylene quinuclidinone (MQ), which binds covalently to the p53 core domain (Figure 3). Cys277 is a prime binding site for MQ in p53 and is essential for MQ-mediated thermostabilization of R175H- and R273H-mutant p53, converting the protein to a WT p53–like conformation and exhibiting WT p53 activity. Importantly, both Cys124 and Cys277 are required for eprenetapopt-mediated R175H-mutant p53 reactivation (89–91).

In addition, eprenetapopt has been shown to have alternative mechanisms to induce cell death, such as eprenetapopt's reaction with other thiol group–containing cellular molecules. Thus, eprenetapopt has been reported to attach to and deplete thiol-containing GSH, resulting in increased ROS (92–95). The ability of eprenetapopt to increase ROS levels may contribute to anticancer activity observed in WT p53 and p53-depleted cancer cells (71, 90, 96, 97). Along with the ability to reactivate mutant p53 and generate ROS, eprenetapopt exhibited potent antitumor activity in a wide range of preclinical cancer models in vitro and in vivo (71, 90, 96).

A phase Ib/II study of the combination of eprenetapopt and AZA in 45 patients with *TP53*-mutant myelodysplastic syndromes or AML showed a favorable toxicity profile and led to clinical responses in 71% of patients, including complete responses in 44%. However, the combination of eprenetapopt plus AZA failed to significantly increase the rate of complete responses in a phase III trial in *TP53*-mutant myelodysplastic syndromes, ending the clinical development of this drug (98) (Table 2).

KG13. Besides mutations on the DNA-binding domain (DBD) of p53, Y220C is the most common cancerous mutation and is responsible for approximately 100,000 cancer cases per year worldwide (99). It creates a cavity on the surface of p53 and also leads to loss of DNA-binding ability at room temperature (72). The small molecule PhiKan083 is a lead compound that is able to insert into the Y220C pocket (100, 101) ~~a mutation that indirectly inhibits DNA binding through the loss of thermal stability in the DBD at room temperature (72). The compound PhiKan083 is a carbazole derivative (100) that was subsequently developed to bind within the p53 Y220C cavity and has undergone chemical modification to improve both affinity and thermal stabilization of mutant p53 (101)~~ (Figure 2). Although the PhiKan compounds have demonstrated the potential to target p53 Y220C, none of them satisfy the potency requirements of drug candidates because PhiKan are reversible binding compounds (72). KG13, an azaindole derivative (72), selectively and covalently attaches to the cysteine of mutant p53 Y220C. In Guiley and Shokat's initial characterization of this small molecule, KG13 restored WT p53 thermal stability of the mutant p53 (Figure 3). KG13 restored p53 Y220C DNA-binding ability and led to the expression of proapoptotic p53 target genes, holding potential as a specific therapy for p53 Y220C-mutated cancers ~~KG13-treated cells displayed p53 Y220C–dependent activation of p53 target genes with growth inhibition and increased caspase~~

~~activity~~ (72). ~~To our knowledge, KG13 is the first allele-specific compound that selectively reacts with the cysteine p53 Y220C to rescue WT p53 thermal stability and gene activation. Similarly to sotorasib, the KRAS G12C covalent inhibitor, the reactivity of KG13 toward the p53 somatic mutant cysteine Y220C provides a precision-medicine approach to generating WT p53 activity specifically in tumor cells harboring the p53 Y220C mutation.~~

Novel compounds causing depletion of mutant p53

Depletion of mutant p53 prevents both mutant p53 gain-of-function and dominant-negative effects. HSP90 is an ATP-dependent molecular chaperone that reversibly binds to and stabilizes p53. Ganetespib binds to the ATP-binding domain of HSP90, inhibiting the ATPase activity of the HSP90 core protein (102, 103). Ganetespib potently inhibited cancer cell proliferation in vitro and in human tumor xenografts in multiple types of cancer (102–105). However, these studies did not address whether ganetespib's effects are relevant to WT or mutant p53. SAHA (vorinostat) is an FDA-approved inhibitor of class I, II, and IV histone deacetylases (HDACs) and epigenetically regulates the malignant properties of multiple cancer types (106). Mutant p53s are stabilized by forming an HDAC6/HSP90/mutant p53 complex in cancer cells (107–110) (Figure 3). Alexandrova et al. reported that genetic and pharmacological depletion of mutant p53 (R248Q) by ganetespib or SAHA inhibits the growth of human breast MDA-MB-231 cancer cells in a mutant p53-dependent manner (107–109). In $p53^{R172H/R172H}$ and $p53^{R248Q/-}$ mice, ganetespib treatment inhibited tumor growth and extended survival, which was not observed in control $p53^{-/-}$ mice (107). Ganetespib was investigated in phase I/II clinical trials in combination with paclitaxel for the treatment of p53-mutated platinum-resistant ovarian cancers, and it did not improve patient outcomes (111). Despite negative results in ovarian cancer, the clinical activity of ganetespib in other p53-mutated tumors as monotherapy or in combination with other agents remains unknown. Zhang et al. reported that compound NSC59984 induces mutant p53 degradation through activation of MDM2 and stimulates p73 activity, leading to p73-mediated cell apoptosis in p53-mutated CRC cells (76) (Figure 3).

Targeting the ISR in cancer therapy

ISR is a conserved signaling pathway in eukaryotic cells that is activated in response to a range of physiological changes and different pathological conditions. The ISR is triggered by eIF2α phosphorylation that is mediated by the eIF2α kinases PERK, GCN2, HRI, and PKR. The kinases are activated by different stressors, such as ER stress (PERK), amino acid deficiency (GCN2), heme decrease (HRI), and dsRNA virus infection (PKR) (112). ~~ER stress, amino acid deprivation, glucose deprivation, heme downregulation, and viral infection all constitute stressful stimuli that activate the ISR phosphorylation of the α subunit of eukaryotic translation initiation factor 2 (eIF2α) at serine 51. In mammalian cells, this is catalyzed by a family of four serine/threonine (S/T) eIF2α kinases (PERK, GCN2, PKR, and HRI) that are activated by distinct stress stimuli.~~ We will focus on proteasome inhibitors and imipridones, which activate PERK and HRI, respectively (Figure 4). eIF2α phosphorylation inhibits 5′-cap–dependent protein translation, which selectively activates the translation of mRNAs with uORFs in the 5′-UTR region, such as ATF4 (112, 113), ~~eIF2α phosphorylation causes reduction in global protein synthesis while allowing the translation of selected genes including ATF4,~~ a basic leucine zipper (bZIP) transcription factor belonging to the ATF/CREB family (113~~2~~). ATF4 regulates expression of its target genes to help cell survival and recovery. Due to rapid growth, proliferation, and hypoxia in tumor microenvironment, cancer cells often exhibit an elevated ISR compared to normal cells, and its upregulation in cancer cells contributes to their survival, growth, and resistance to drug treatments. ~~Cancer cells may elevate the protective effects of the ISR to facilitate survival during conditions of stress associated with rapid growth, proliferation, and hypoxia and to evade programmed cell death.~~ However, if the cellular stress is severe, either in intensity or in duration, ATF4 regulates the expression of another set of genes to execute cell death (114~~3~~–116~~5~~) (Figure 4).

ATF4 is a key effector of cell fate in response to the ISR. When ATF4 is not bound to its DNA target, it exists as a monomer (117~~6~~). ATF4 can interact with bZIP or AP-1 transcription factors to form heterodimers. Transcriptional selectivity of ATF4 is modulated by the formation of heterodimers with CHOP or ATF3, both of which are transcriptional targets of ATF4. ATF4-ATF3 heterodimers regulate various cellular processes, including stress responses, apoptosis, and autophagy. ATF4-CHOP heterodimers mainly regulate apoptosis (112, 118–120). ~~For example, interactions with ATF3 enhance cellular efforts to reestablish homeostasis, while interactions with CHOP promote cell apoptosis (117, 118) or autophagy (119).~~

ATF4 regulates CHOP gene expression, which is important for mediating ISR-induced apoptosis. ~~One of the best studied mechanisms of ISR-induced cell apoptosis is through ATF4-mediated activation of CHOP.~~ CHOP is a transcription factor belonging to the bZIP family. CHOP induces apoptosis by upregulating BIM, PUMA, NOXA, and DR5, affecting both the intrinsic and extrinsic pathways (114~~3~~, 115~~4~~). ATF4 itself can promote apoptosis by directly upregulating NOXA and PUMA expression, leading to cancer cell apoptosis (75, 121~~0~~, 122~~1~~). Also, ATF4 promotes XIAP protein degradation through the ubiquitin-proteasome system, ensuring apoptosis together with CHOP upregulation (123~~2~~). CHOP-ATF3 heterodimers can increase the transcription of DR5, thus promoting apoptosis (124~~3~~). ATF4-CHOP heterodimers regulate the expression of proapoptotic genes such as *PUMA*, *NOXA*, and *APAF1* (125~~4~~, 126~~5~~).

ONC201

ONC201 is a first-in-class imipridone compound that is currently being tested in clinical trials for various malignancies, including leukemia, lymphoma, and colon, prostate, breast, and CNS tumors ~~has emerged as a promising drug candidate for treating a diverse range of solid and hematologic cancers~~ (127~~6~~). The drug was originally discovered as a TRAIL-inducing compound (TIC10) in a chemical library screen and was shown to inhibit cancer cell viability (128~~7~~). The most well-characterized imipridones include ONC201, ONC206, and ONC212. The clinical trials demonstrate that ONC201 is a well-tolerated and potent anticancer drug in previously treatment-resistant patients (129). ONC201 shows potent inhibitory effects on H3K27M-mutated diffuse midline glioma (DMG) ~~ONC201 exhibits cytotoxicity~~

~~across a spectrum of preclinical cancer models and has entered phase 1 and 2 clinical trials for treating patients with leukemia, lymphoma and colon, prostate, breast, and CNS tumors (126). ONC201 has demonstrated a favorable safety profile and encouraging antitumor activity in patients with advanced treatment-refractory solid tumors (128). In addition, ONC201 demonstrates CNS tumor penetration and encouraging response rates in a subset of both adult and pediatric brain cancer patients with H3K27M-mutant diffuse midline glioma (DMG)~~ (130~~29~~–133~~2~~). The encouraging preliminary clinical activity in DMG led to an ongoing international, randomized phase III trial with ONC201 for the treatment of newly diagnosed H3 K27M–mutant diffuse glioma following completion of radiotherapy (NCT05580562). Another trial is investigating ONC206 in adults with recurrent primary CNS tumors (NCT04732065) (Table 2).

As mentioned above, ONC201 was originally called TRAIL-inducing compound 10 (TIC10) and was later discovered to activate the ISR, causing cell death through upregulation of the TRAIL/DR5 extrinsic pathway and ATF4 (128~~7~~, 134~~3~~). Studies have indicated multiple pathways as putative mechanisms, including dopamine receptor antagonism, activation of the TRAIL-mediated extrinsic pathway, and regulation of the ISR. Here, we focus on the ISR-mediated effects of imipridones. In an effort to search for the direct targets of imipridones, ONC201 and ONC212 were found to act as potent activators of caseinolytic mitochondrial matrix peptidase proteolytic subunit (ClpP) (135~~4~~, 136~~5~~). ClpP localizes to the mitochondrial matrix and is essential for homeostasis of mitochondrial proteins. ClpX acts as a substrate-recognizing subunit for the ClpP protease, forming the ClpXP complex. This complex is essential for protein degradation ~~ClpP activity is tightly regulated by ClpX, which specifically recognizes and unfolds its substrates, then feeds them into ClpP's proteolytic chamber for degradation~~ (137~~6~~) (Figure 4).

The crystal structure of the ONC201-ClpP complex indicates that ONC201 binds to the hydrophobic pockets between adjacent ClpP subunits. This binding disrupts the protein-protein interaction between ClpP and ClpX and induces opening of ClpP's axial entrance pore, which is normally opened by ClpX. ONC201 causes ClpP's entrance pore radius to enlarge from 12 to 17Å. As a result, ONC201 activates ClpP in the absence of ClpX (135~~4~~, 136~~5~~). Activated ClpP cleaves many mitochondrial proteins, including those required for oxidative phosphorylation, resulting in mitochondrial stress, leading to activation of the ISR and ATF4 upregulation (135~~4~~, 136~~5~~) (Figure 4). But the mechanism connecting ClpP activation to ATF4 upregulation still is unknown. The ONC201 analog, ONC212, has a highly electronegative p-CF3 benzyl substituent that extends into ClpP's apolar pocket and enhances affinity with the protease (136~~5~~). That enhanced affinity is consistent with the observation that ONC212 is about 10-fold more potent than ONC201.

Imipridone treatment induces gene-expression profiles consistent with ISR activation, mainly by upregulating the expression of ATF4 (134~~3~~) Imipridones trigger typical or atypical ISR, depending on cancer cell type. For example, in colorectal (134), AML (138), and breast cancer cells (139), imipridones induce typical ISR. On the other hand, in MCL (138) and cutaneous T cell lymphoma (CTCL) (140), imipridones activate atypical ISR. ~~Interestingly, imipridones can activate either the typical or atypical ISR in a cell type–specific way. Typical ISR pathway activation is observed in preclinical models of AML (137), colorectal (133), and breast (138) cancer. In contrast, in mantle cell lymphoma (MCL) (137) and cutaneous T cell lymphoma (CTCL) (139), imipridone treatment activates ATF4 through an atypical, phospho-eIF2α–independent manner.~~ The mechanisms of atypical ISR activation also remain elusive.

Bortezomib and carfilzomib

The proteasome is a large protease complex that degrades many cellular proteins via a ubiquitin-dependent system (141~~0~~, 142~~1~~). MM is an incurable clonal B cell malignancy characterized by the accumulation of terminally differentiated, antibody-producing plasma cells in the bone marrow (143~~2~~). Bortezomib was the first-in-class compound to be approved by the FDA for MM and is a cornerstone of antimyeloma therapy (144~~3~~, 145~~4~~). Carfilzomib is a second-generation proteasome inhibitor with an improved efficacy and safety profile compared with bortezomib (146~~5~~) (Table 2).

Bortezomib is a reversible inhibitor of the proteasome with a peptide-like backbone and boronated group. In contrast, carfilzomib is an irreversible proteasome inhibitor that contains an epoxyketone as an active group (146~~5~~). Inhibition of the proteasome leads to the accumulation of polyubiquitinated misfolded or unfolded proteins (PUMUP), which leads to ER stress and upregulation of ATF4 through the ISR. Thus, ATF4-mediated apoptosis is an important mechanism of proteasome inhibitors (147~~6~~, 148~~7~~) (Figure 4). However, acquired or secondary resistance consistently emerges in patients who initially respond to proteasome inhibitors (149~~8~~). Two resistance mechanisms have been identified (Figure 4). Inhibition of the proteasome promotes the degradation of unfolded and misfolded proteins through the aggresome pathway, which relieves the accumulation of unwanted proteins and the ISR (147~~6~~, 150~~49~~). Polyubiquitinated proteins (PUPs) in association with HDAC6 bind to dynein motor protein. The PUP-HDAC6-dynein complex moves to the aggresome along the microtubule. Aggresome formation ultimately induces autophagic clearance, which terminates in lysosomal degradation (147~~6~~, 150~~49~~). Therefore, the dual inhibition of HDAC6 and the proteasome triggers dramatic and prolonged accumulation of unwanted proteins and induces apoptosis in resistant myeloma cells (RPMI-8226v10r, Kas6v10r, RPMI-LR5, and RPMI-Dox40) (147~~6~~, 151~~0~~, 152~~1~~). ER stress induced by proteasome inhibitors can also promote HDAC4 binding to ATF4 to prevent its nuclear translocation, hence inhibiting ATF4 transcriptional activity and leading to cells resistant to bortezomib or carfilzomib treatments (152~~1~~–154~~3~~). Dual inhibition of HDAC4 and proteasome synergistically activates ATF4-mediated cell apoptosis (153~~2~~–155~~4~~).

PG3-Oc and CB002 preclinical development

The third approach mentioned above aims at restoring expression of proapoptotic p53 target genes in a p53-independent way in p53-deficient tumors. These approaches may be broadly applicable, as WT p53, p53-deleted, and p53-mutated tumors could all be targeted. Compound PG3-Oc is an analogue of the natural product prodigiosin, and it triggers ISR and leads to activation of ATF4 (Figure 4). ATF4 regulates the expression of a subset of p53

target genes in p53-deficient HCT116$^{-/-}$ and p53-mutated HT29 cells, including *PUMA*, *DR5*, *NOXA*, and *CDKN1A* (encoding p21). Among them, PUMA plays an important role in mediating cancer cell apoptosis (75).

CB002 and its derivatives are xanthine analogs. They induce ISR and ATF4-mediated expression of NOXA and DR5 (Figure 4). NOXA is responsible for cell apoptosis (74). Transcriptomic and proteomic analyses show that PG3-Oc and CB002 upregulate transcriptomes and proteomes that overlap with the p53 target gene database. Importantly, the overlapping gene sets contain typical p53 target genes that regulate cell cycle and apoptosis as mentioned above. Although p53 and ATF4 generally control different genes, they converge on a set of common transcriptional targets related to apoptosis. A recent paper studied shared gene targets of ATF4 and p53 transcriptional networks (156~~5~~). Authors report that the p53 and ISR pathways converge to independently regulate common metabolic and proapoptotic genes. They demonstrate that these targets require p53 during DNA-damage response, but not during the ISR. In contrast, ATF4 is required during the ISR and is dispensable under p53-activating conditions (156~~5~~). These results provide a rationale for combined treatments of DNA-damaging drugs or MDM2 inhibitors with ISR inducers to achieve synergistic antitumor effects in WT p53 tumors. Andrysik et al. reported that inhibition of the phosphatase PPM1D led to activation of ATF4 through ISR (157~~6~~). Nelfinavir is an inhibitor of HIV-1 protease and a robust ISR inducer (158~~7~~). PPM1D inhibitor or nelfinavir synergized with MDM2 inhibitors to amplify expression of some p53 targets and synergistically increase cell death in vitro and in HCT116 tumor xenografts (157~~6~~).

Conclusions

Dysregulation of and resistance to apoptosis is a hallmark of cancer cells due to mutations in the extrinsic, intrinsic, p53, and ISR pathways. Targeting these apoptotic pathways is an intriguing approach to identifying new antitumor therapies. The ability to target and activate apoptosis in resistant tumor cells will continue to evolve in future clinical practice. The future development of agents that target apoptotic pathways either directly or indirectly through the p53 and ISR pathways could lead to disease regression or cures in patients with difficult-to-treat tumors.

Acknowledgments

WSED is an American Cancer Society Research Professor and is supported by the Mencoff Family University Professorship at Brown University.

Address correspondence to: Wafik S. El-Deiry, Warren Alpert Medical School, Brown University, Department of Pathology and Laboratory Medicine, Campus Box G-E5, 70 Ship Street, Room 537, Providence, Rhode Island 02903, USA. Email: wafik@brown.edu. Or to: Xiaobing Tian, Department of Pathology and Laboratory Medicine, Warren Alpert Medical School, Brown University 70 Ship Street, Room 527A, Providence, Rhode Island 02903, USA. Email: xiaobing_tian@brown.edu.

1. Singh R, et al. Regulation of apoptosis in health and disease: the balancing act of BCL-2 family proteins. *Nat Rev Mol Cell Biol*. 2019;20(3):175–193.
2. Lopez J, Tait SW. Mitochondrial apoptosis: killing cancer using the enemy within. *Br J Cancer*. 2015;112(6):957–962.
3. Birkinshaw RW, Czabotar PE. The BCL-2 family of proteins and mitochondrial outer membrane permeabilisation. *Semin Cell Dev Biol*. 2017;72:152–162.
4. Czabotar PE, et al. Control of apoptosis by the BCL-2 protein family: implications for physiology and therapy. *Nat Rev Mol Cell Biol*. 2014;15(1):49–63.
5. Kim H, et al. Stepwise activation of BAX and BAK by tBID, BIM, and PUMA initiates mitochondrial apoptosis. *Mol Cell*. 2009;36(3):487–499.
6. Letai A, et al. Distinct BH3 domains either sensitize or activate mitochondrial apoptosis, serving as prototype cancer therapeutics. *Cancer Cell*. 2002;2(3):183–192.
7. Du H, et al. BH3 domains other than Bim and Bid can directly activate Bax/Bak. *J Biol Chem*. 2011;286(1):491–501.
8. Hockings C, et al. Bid chimeras indicate that most BH3-only proteins can directly activate Bak and Bax, and show no preference for Bak versus Bax. *Cell Death Dis*. 2015;6(4):e1735.
9. Chaudhary PM, et al. Death receptor 5, a new member of the TNFR family, and DR4 induce FADD-dependent apoptosis and activate the NF-kappaB pathway. *Immunity*. 1997;7(6):821–830.
10. Guicciardi ME, Gores GJ. Life and death by death receptors. *FASEB J*. 2009;23(6):1625–1637.
11. Wu GS, et al. KILLER/DR5 is a DNA damage-inducible p53-regulated death receptor gene. *Nat Genet*. 1997;17(2):141–143.
12. Di Cristofano F, et al. Therapeutic targeting of TRAIL death receptors. *Biochem Soc Trans*. 2023;51(1):57–70.
13. Irmler M, et al. Inhibition of death receptor signals by cellular FLIP. *Nature*. 1997;388(6638):190–195.
14. Ozoren N, El-Deiry WS. Defining characteristics of Types I and II apoptotic cells in response to TRAIL. *Neoplasia*. 2002;4(6):551–557.
15. Gillissen B, et al. Endogenous Bak inhibitors Mcl-1 and Bcl-xL: differential impact on TRAIL resistance in Bax-deficient carcinoma. *J Cell Biol*. 2010;188(6):851–862.
16. Du C, et al. Smac, a mitochondrial protein that promotes cytochrome c-dependent caspase activation by eliminating IAP inhibition. *Cell*. 2000;102(1):33–42.
17. Vogler M, et al. Targeting XIAP bypasses Bcl-2-mediated resistance to TRAIL and cooperates with TRAIL to suppress pancreatic cancer growth in vitro and in vivo. *Cancer Res*. 2008;68(19):7956–7965.
18. Nechiporuk T, et al. The TP53 apoptotic network is a primary mediator of resistance to BCL2 inhibition in AML Cells. *Cancer Discov*. 2019;9(7):910–925.
19. Kumar R, et al. Cytochrome *c* deficiency confers apoptosome and mitochondrial dysfunction in african-american men with prostate cancer. *Cancer Res*. 2019;79(7):1353–1368.
20. Yadav N, et al. Molecular insights on cytochrome c and nucleotide regulation of apoptosome function and its implication in cancer. *Biochim Biophys Acta Mol Cell Res*. 2020;1867(1):118573.
21. Koornstra JJ, et al. Expression of TRAIL (TNF-related apoptosis-inducing ligand) and its receptors in normal colonic mucosa, adenomas, and carcinomas. *J Pathol*. 2003;200(3):327–335.
22. Micheau O, et al. The long form of FLIP is an activator of caspase-8 at the Fas death-inducing signaling complex. *J Biol Chem*. 2002;277(47):45162–45171.
23. Singh N, et al. Impaired death receptor signaling in leukemia causes antigen-independent resistance by inducing CAR T-cell dysfunction. *Cancer Discov*. 2020;10(4):552–567.
24. Stolfi C, et al. Molecular targets of TRAIL-sensitizing agents in colorectal cancer. *Int J Mol Sci*. 2012;13(7):7886–7901.
25. Carneiro BA, El-Deiry WS. Targeting apoptosis in cancer therapy. *Nat Rev Clin Oncol*. 2020;17(7):395–417.
26. Roberts AW, et al. Targeting BCL2 with venetoclax in relapsed chronic lymphocytic leukemia. *N Engl J Med*. 2016;374(4):311–322.
27. Stilgenbauer S, et al. Venetoclax for patients with chronic lymphocytic leukemia with 17p deletion: results from the full population of a phase II pivotal trial. *J Clin Oncol*. 2018;36(19):1973–1980.
28. Khaw SL, et al. Both leukaemic and normal peripheral B lymphoid cells are highly sensitive to the selective pharmacological inhibition of prosurvival Bcl-2 with ABT-199. *Leukemia*. 2014;28(6):1207–1215.
29. Fischer K, et al. Venetoclax and obinutuzumab in

patients with CLL and coexisting conditions. *N Engl J Med*. 2019;380(23):2225–2236.
30. DiNardo CD, et al. Azacitidine and venetoclax in previously untreated acute myeloid leukemia. *N Engl J Med*. 2020;383(7):617–629.
31. Wei AH, et al. Venetoclax plus LDAC for newly diagnosed AML ineligible for intensive chemotherapy: a phase 3 randomized placebo-controlled trial. *Blood*. 2020;135(24):2137–2145.
32. Hymowitz SG, et al. Triggering cell death: the crystal structure of Apo2L/TRAIL in a complex with death receptor 5. *Mol Cell*. 1999;4(4):563–571.
33. LeBlanc HN, Ashkenazi A. Apo2L/TRAIL and its death and decoy receptors. *Cell Death Differ*. 2003;10(1):66–75.
34. Merchant MS, et al. Phase I trial and pharmacokinetic study of lexatumumab in pediatric patients with solid tumors. *J Clin Oncol*. 2012;30(33):4141–4147.
35. Mom CH, et al. Mapatumumab, a fully human agonistic monoclonal antibody that targets TRAIL-R1, in combination with gemcitabine and cisplatin: a phase I study. *Clin Cancer Res*. 2009;15(17):5584–5590.
36. Plummer R, et al. Phase 1 and pharmacokinetic study of lexatumumab in patients with advanced cancers. *Clin Cancer Res*. 2007;13(20):6187–6194.
37. von Pawel J, et al. Phase II trial of mapatumumab, a fully human agonist monoclonal antibody to tumor necrosis factor-related apoptosis-inducing ligand receptor 1 (TRAIL-R1), in combination with paclitaxel and carboplatin in patients with advanced non-small-cell lung cancer. *Clin Lung Cancer*. 2014;15(3):188–196.
38. Herbst RS, et al. Phase I dose-escalation study of recombinant human Apo2L/TRAIL, a dual proapoptotic receptor agonist, in patients with advanced cancer. *J Clin Oncol*. 2010;28(17):2839–2846.
39. Soria JC, et al. Phase 1b study of dulanermin (recombinant human Apo2L/TRAIL) in combination with paclitaxel, carboplatin, and bevacizumab in patients with advanced non-squamous non-small-cell lung cancer. *J Clin Oncol*. 2010;28(9):1527–1533.
40. Naval J, et al. Importance of TRAIL molecular anatomy in receptor oligomerization and signaling. Implications for cancer therapy. *Cancers (Basel)*. 2019;11(4):444.
41. Papadopoulos KP, et al. Unexpected hepatotoxicity in a phase I study of TAS266, a novel tetravalent agonistic Nanobody® targeting the DR5 receptor. *Cancer Chemother Pharmacol*. 2015;75(5):887–895.
42. Kim TH, et al. PEGylated TNF-related apoptosis-inducing ligand (TRAIL) analogues: pharmacokinetics and antitumor effects. *Bioconjug Chem*. 2011;22(8):1631–1637.
43. Chae SY, et al. Improved antitumor activity and tumor targeting of NH(2)-terminal-specific PEGylated tumor necrosis factor-related apoptosis-inducing ligand. *Mol Cancer Ther*. 2010;9(6):1719–1729.
44. Hamacher R, et al. Apoptotic pathways in pancreatic ductal adenocarcinoma. *Mol Cancer*. 2008;7:64.
45. Hinz S, et al. Bcl-XL protects pancreatic adenocarcinoma cells against CD95- and TRAIL-receptor-mediated apoptosis. *Oncogene*. 2000;19(48):5477–5486.
46. Deveraux QL, Reed JC. IAP family proteins — suppressors of apoptosis. *Genes Dev*. 1999;13(3):239–252.
47. Jhaveri AV, et al. Combination of ONC201 and TLY012 induces selective, synergistic apoptosis in vitro and significantly delays PDAC xenograft growth in vivo. *Cancer Biol Ther*. 2021;22(10–12):607–618.
48. Louie AD, et al. Combining PD-1 inhibition with the TRAIL receptor agonist TLY012 promotes pancreatic ductal adenocarcinoma tumor regression in an immunocompetent mouse model. *Cancer Res*. 2022;82(12_suppl):5607.
49. Doi T, et al. Phase 1 study of conatumumab, a pro-apoptotic death receptor 5 agonist antibody, in Japanese patients with advanced solid tumors. *Cancer Chemother Pharmacol*. 2011;68(3):733–741.
50. Forero-Torres A, et al. Phase I trial of weekly tigatuzumab, an agonistic humanized monoclonal antibody targeting death receptor 5 (DR5). *Cancer Biother Radiopharm*. 2010;25(1):13–19.
51. Herbst RS, et al. A first-in-human study of conatumumab in adult patients with advanced solid tumors. *Clin Cancer Res*. 2010;16(23):5883–5891.
52. Soria JC, et al. Randomized phase II study of dulanermin in combination with paclitaxel, carboplatin, and bevacizumab in advanced non-small-cell lung cancer. *J Clin Oncol*. 2011;29(33):4442–4451.
53. Subbiah V, et al. Targeting the apoptotic pathway in chondrosarcoma using recombinant human Apo2L/TRAIL (dulanermin), a dual proapoptotic receptor (DR4/DR5) agonist. *Mol Cancer Ther*. 2012;11(11):2541–2546.
54. Trarbach T, et al. Phase II trial of mapatumumab, a fully human agonistic monoclonal antibody that targets and activates the tumour necrosis factor apoptosis-inducing ligand receptor-1 (TRAIL-R1), in patients with refractory colorectal cancer. *Br J Cancer*. 2010;102(3):506–512.
55. Gieffers C, et al. APG350 induces superior clustering of TRAIL receptors and shows therapeutic antitumor efficacy independent of cross-linking via Fcγ receptors. *Mol Cancer Ther*. 2013;12(12):2735–2747.
56. Wilson NS, et al. An Fcγ receptor-dependent mechanism drives antibody-mediated target-receptor signaling in cancer cells. *Cancer Cell*. 2011;19(1):101–113.
57. Phillips DC, et al. Hexavalent TRAIL fusion protein eftozanermin alfa optimally clusters apoptosis-inducing TRAIL receptors to induce on-target antitumor activity in solid tumors. *Cancer Res*. 2021;81(12):3402–3414.
58. LoRusso P, et al. Eftozanermin alfa (ABBV-621) monotherapy in patients with previously treated solid tumors: findings of a phase 1, first-in-human study. *Invest New Drugs*. 2022;40(4):762–772.
59. Huet HA, et al. Multivalent nanobodies targeting death receptor 5 elicit superior tumor cell killing through efficient caspase induction. *MAbs*. 2014;6(6):1560–1570.
60. Subbiah V, et al. Preclinical characterization and Phase I Trial Results of INBRX-109, a third-generation, recombinant, humanized, death receptor 5 agonist antibody, in chondrosarcoma. *Clin Cancer Res*. 2023;29(16):2988–3003.
61. Lane DP. Cancer. p53, guardian of the genome. *Nature*. 1992;358(6381):15–16.
62. Takimoto R, El-Deiry WS. Wild-type p53 transactivates the KILLER/DR5 gene through an intronic sequence-specific DNA-binding site. *Oncogene*. 2000;19(14):1735–1743.
63. Liu X, et al. p53 upregulates death receptor 4 expression through an intronic p53 binding site. *Cancer Res*. 2004;64(15):5078–5083.
64. Kuribayashi K, et al. TNFSF10 (TRAIL), a p53 target gene that mediates p53-dependent cell death. *Cancer Biol Ther*. 2008;7(12):2034–2038.
65. Aubrey BJ, et al. How does p53 induce apoptosis and how does this relate to p53-mediated tumour suppression? *Cell Death Differ*. 2018;25(1):104–113.
66. Chen J. The cell-cycle arrest and apoptotic functions of p53 in tumor initiation and progression. *Cold Spring Harb Perspect Med*. 2016;6(3):a026104.
67. Tuval A, et al. Pharmacological reactivation of p53 in the era of precision anticancer medicine. *Nat Rev Clin Oncol*. 2024;21(2):106–120.
68. Aziz MH, et al. Acquisition of p53 mutations in response to the non-genotoxic p53 activator Nutlin-3. *Oncogene*. 2011;30(46):4678–4686.
69. Kocik J, et al. Helping the released guardian: drug combinations for supporting the anticancer activity of HDM2 (MDM2) antagonists. *Cancers (Basel)*. 2019;11(7):1014.
70. Montesinos P, et al. MIRROS: a randomized, placebo-controlled, Phase III trial of cytarabine ± idasanutlin in relapsed or refractory acute myeloid leukemia. *Future Oncol*. 2020;16(13):807–815.
71. Bykov VJN, et al. Targeting mutant p53 for efficient cancer therapy. *Nat Rev Cancer*. 2018;18(2):89–102.
72. Guiley KZ, Shokat KM. A small molecule reacts with the p53 somatic mutant Y220C to rescue wild-type thermal stability. *Cancer Discov*. 2023;13(1):56–69.
73. Nishikawa S, Iwakuma T. Drugs targeting p53 mutations with FDA approval and in clinical trials. *Cancers (Basel)*. 2023;15(2):429.
74. Hernandez Borrero L, et al. A subset of CB002 xanthine analogs bypass p53-signaling to restore a p53 transcriptome and target an S-phase cell cycle checkpoint in tumors with mutated-p53. *Elife*. 2021;10:e70429.
75. Tian X, et al. P53-independent partial restoration of the p53 pathway in tumors with mutated p53 through ATF4 transcriptional modulation by ERK1/2 and CDK9. *Neoplasia*. 2021;23(3):304–325.
76. Zhang S, et al. Small-molecule NSC59984 restores p53 pathway signaling and antitumor effects against colorectal cancer via p73 activation and degradation of mutant p53. *Cancer Res*. 2015;75(18):3842–3852.
77. Chene P. Inhibiting the p53-MDM2 interaction: an important target for cancer therapy. *Nat Rev Cancer*. 2003;3(2):102–109.
78. Wasylishen AR, Lozano G. Attenuating the p53 pathway in human cancers: many means to the same end. *Cold Spring Harb Perspect Med*. 2016;6(8):a026211.
79. Liao G, et al. The development of piperidinones

as potent MDM2-P53 protein-protein interaction inhibitors for cancer therapy. *Eur J Med Chem*. 2018;159:1–9.

80. Erba HP, et al. Phase 1b study of the MDM2 inhibitor AMG 232 with or without trametinib in relapsed/refractory acute myeloid leukemia. *Blood Adv*. 2019;3(13):1939–1949.
81. Furet P, et al. Discovery of a novel class of highly potent inhibitors of the p53-MDM2 interaction by structure-based design starting from a conformational argument. *Bioorg Med Chem Lett*. 2016;26(19):4837–4841.
82. Fang DD, et al. MDM2 inhibitor APG-115 synergizes with PD-1 blockade through enhancing antitumor immunity in the tumor microenvironment. *J Immunother Cancer*. 2019;7(1):327.
83. Fang DD, et al. MDM2 inhibitor APG-115 exerts potent antitumor activity and synergizes with standard-of-care agents in preclinical acute myeloid leukemia models. *Cell Death Discov*. 2021;7(1):90.
84. Arena G, et al. Mitochondrial MDM2 Regulates Respiratory Complex I Activity Independently of p53. *Mol Cell*. 2018;69(4):594–609.
85. Arena G, et al. MDM2 controls gene expression independently of p53 in both normal and cancer cells. *Cell Death Differ*. 2018;25(9):1533–1535.
86. Cao Z, et al. MDM2 promotes genome instability by ubiquitinating the transcription factor HBP1. *Oncogene*. 2019;38(24):4835–4855.
87. Muller PA, Vousden KH. Mutant p53 in cancer: new functions and therapeutic opportunities. *Cancer Cell*. 2014;25(3):304–317.
88. Alvarado-Ortiz E, et al. Mutant p53 Gain-of-function: role in cancer development, progression, and therapeutic approaches. *Front Cell Dev Biol*. 2020;8:607670.
89. Hassin O, Oren M. Drugging p53 in cancer: one protein, many targets. *Nat Rev Drug Discov*. 2023;22(2):127–144.
90. Perdrix A, et al. PRIMA-1 and PRIMA-1^{Met} (APR-246): from mutant/wild type p53 reactivation to unexpected mechanisms underlying their potent anti-tumor effect in combinatorial therapies. *Cancers (Basel)*. 2017;9(12):172.
91. Zhang Q, et al. APR-246 reactivates mutant p53 by targeting cysteines 124 and 277. *Cell Death Dis*. 2018;9(5):439.
92. Liu DS, et al. Inhibiting the system x_C^-/glutathione axis selectively targets cancers with mutant-p53 accumulation. *Nat Commun*. 2017;8:14844.
93. Mohell N, et al. APR-246 overcomes resistance to cisplatin and doxorubicin in ovarian cancer cells. *Cell Death Dis*. 2015;6(6):e1794.
94. Ogiwara H, et al. Targeting the vulnerability of glutathione metabolism in ARID1A-deficient cancers. *Cancer Cell*. 2019;35(2):177–190.
95. Tessoulin B, et al. PRIMA-1Met induces myeloma cell death independent of p53 by impairing the GSH/ROS balance. *Blood*. 2014;124(10):1626–1636.
96. Duffy MJ, et al. Mutant p53 as a target for cancer treatment. *Eur J Cancer*. 2017;83:258–265.
97. Patyka M, et al. Sensitivity to PRIMA-1MET is associated with decreased MGMT in human glioblastoma cells and glioblastoma stem cells irrespective of p53 status. *Oncotarget*. 2016;7(37):60245–60269.
98. Louie AD, et al. Combining PD-1 inhibition with the TRAIL receptor agonist TLY012 promotes pancreatic ductal adenocarcinoma tumor regression in an immunocompetent mouse model. *Cancer Res*. 2022;82(12_suppl):5607.
99. Sundar D, et al. Wild type p53 function in $p53^{Y220C}$ mutant harboring cells by treatment with Ashwagandha derived anticancer withanolides: bioinformatics and experimental evidence. *J Exp Clin Cancer Res*. 2019;38(1):103.
100. Boeckler FM, et al. Targeted rescue of a destabilized mutant of p53 by an in silico screened drug. *Proc Natl Acad Sci U S A*. 2008;105(30):10360–10365.
101. Bauer MR, et al. Targeting cavity-creating p53 cancer mutations with small-molecule stabilizers: the Y220X paradigm. *ACS Chem Biol*. 2020;15(3):657–668.
102. Shimamura T, et al. Ganetespib (STA-9090), a nongeldanamycin HSP90 inhibitor, has potent antitumor activity in in vitro and in vivo models of non-small cell lung cancer. *Clin Cancer Res*. 2012;18(18):4973–4985.
103. Ying W, et al. Ganetespib, a unique triazolone-containing Hsp90 inhibitor, exhibits potent antitumor activity and a superior safety profile for cancer therapy. *Mol Cancer Ther*. 2012;11(2):475–484.
104. Bansal H, et al. Heat shock protein 90 regulates the expression of Wilms tumor 1 protein in myeloid leukemias. *Blood*. 2010;116(22):4591–4599.
105. McCleese JK, et al. The novel HSP90 inhibitor STA-1474 exhibits biologic activity against osteosarcoma cell lines. *Int J Cancer*. 2009;125(12):2792–2801.
106. Marks PA, Xu WS. Histone deacetylase inhibitors: Potential in cancer therapy. *J Cell Biochem*. 2009;107(4):600–608.
107. Alexandrova EM, et al. Improving survival by exploiting tumour dependence on stabilized mutant p53 for treatment. *Nature*. 2015;523(7560):352–356.
108. Li D, et al. SAHA shows preferential cytotoxicity in mutant p53 cancer cells by destabilizing mutant p53 through inhibition of the HDAC6-Hsp90 chaperone axis. *Cell Death Differ*. 2011;18(12):1904–1913.
109. Li D, et al. Functional inactivation of endogenous MDM2 and CHIP by HSP90 causes aberrant stabilization of mutant p53 in human cancer cells. *Mol Cancer Res*. 2011;9(5):577–588.
110. Alexandrova EM, Moll UM. Depleting stabilized GOF mutant p53 proteins by inhibiting molecular folding chaperones: a new promise in cancer therapy. *Cell Death Differ*. 2017;24(1):3–5.
111. Concin N, et al. Phase II results of GANNET53: A European multicenter phase I/randomized II trial of the Hsp90 inhibitor Ganetespib (G) combined with weekly Paclitaxel (P) in women with high-grade serous, high-grade endometrioid, or undifferentiated, platinum-resistant epithelial ovarian, fallopian tube or primary peritoneal cancer. *J Clin Oncol*. 2018;36(15_suppl):5567.
112. Pakos-Zebrucka K, et al. The integrated stress response. *EMBO Rep*. 2016;17(10):1374-95.

113~~2~~. Ameri K, Harris AL. Activating transcription factor 4. *Int J Biochem Cell Biol*. 2008;40(1):14–21.

114~~3~~. Costa-Mattioli M, Walter P. The integrated stress response: From mechanism to disease. *Science*. 2020;368(6489):eaat5314.

115~~4~~. Nwosu GO, et al. Targeting the integrated stress response in hematologic malignancies. *Exp Hematol Oncol*. 2022;11(1):94.

116~~5~~. Tian X, et al. Targeting the integrated stress response in cancer therapy. *Front Pharmacol*. 2021;12:747837.

117~~6~~. Podust LM, et al. Crystal structure of the CCAAT box/enhancer-binding protein beta activating transcription factor-4 basic leucine zipper heterodimer in the absence of DNA. *J Biol Chem*. 2001;276(1):505–513.

118~~7~~. Ohoka N, et al. TRB3, a novel ER stress-inducible gene, is induced via ATF4-CHOP pathway and is involved in cell death. *EMBO J*. 2005;24(6):1243–1255.

119~~8~~. Wang Q, et al. ERAD inhibitors integrate ER stress with an epigenetic mechanism to activate BH3-only protein NOXA in cancer cells. *Proc Natl Acad Sci U S A*. 2009;106(7):2200–2205.

120~~19~~. B'Chir W, et al. The eIF2α/ATF4 pathway is essential for stress-induced autophagy gene expression. *Nucleic Acids Res*. 2013;41(16):7683–7699.

121~~0~~. Armstrong JL, et al. Regulation of endoplasmic reticulum stress-induced cell death by ATF4 in neuroectodermal tumor cells. *J Biol Chem*. 2010;285(9):6091–6100.

122~~1~~. Qing G, et al. ATF4 regulates MYC-mediated neuroblastoma cell death upon glutamine deprivation. *Cancer Cell*. 2012;22(5):631–644.

123~~2~~. Hiramatsu N, et al. Translational and post-translational regulation of XIAP by eIF2α and ATF4 promotes ER stress-induced cell death during the unfolded protein response. *Mol Biol Cell*. 2014;25(9):1411–1420.

124~~3~~. Xu L, et al. PKCδ regulates death receptor 5 expression induced by PS-341 through ATF4-ATF3/CHOP axis in human lung cancer cells. *Mol Cancer Ther*. 2012;11(10):2174–2182.

125~~4~~. Galehdar Z, et al. Neuronal apoptosis induced by endoplasmic reticulum stress is regulated by ATF4-CHOP-mediated induction of the Bcl-2 homology 3-only member PUMA. *J Neurosci*. 2010;30(50):16938–16948.

126~~5~~. Teske BF, et al. CHOP induces activating transcription factor 5 (ATF5) to trigger apoptosis in response to perturbations in protein homeostasis. *Mol Biol Cell*. 2013;24(15):2477–2490.

127~~6~~. Prabhu VV, et al. ONC201 and imipridones: Anti-cancer compounds with clinical efficacy. *Neoplasia*. 2020;22(12):725–744.

128~~7~~. Allen JE, et al. Dual inactivation of Akt and ERK by TIC10 signals Foxo3a nuclear translocation, TRAIL gene induction, and potent antitumor effects. *Sci Transl Med*. 2013;5(171):171ra17.

129~~8~~. Stein MN, et al. First-in-human clinical trial of oral ONC201 in patients with refractory solid tumors. *Clin Cancer Res*. 2017;23(15):4163–4169.

130~~29~~. Arrillaga-Romany I, et al. A phase 2 study of the first imipridone ONC201, a selective DRD2 antagonist for oncology, administered every three weeks in recurrent glioblastoma. *Oncotarget*. 2017;8(45):79298–79304.

131~~0~~. Arrillaga-Romany I, et al. Biological activity of

weekly ONC201 in adult recurrent glioblastoma patients. *Neuro Oncol*. 2020;22(1):94–102.
132~~1~~. Chi AS, et al. Pediatric and adult H3 K27M-mutant diffuse midline glioma treated with the selective DRD2 antagonist ONC201. *J Neurooncol*. 2019;145(1):97–105.
133~~2~~. Hall MD, et al. First clinical experience with DRD2/3 antagonist ONC201 in H3 K27M-mutant pediatric diffuse intrinsic pontine glioma: a case report. *J Neurosurg Pediatr*. 2019;23(6):719–725.
134~~3~~. Kline CL, et al. ONC201 kills solid tumor cells by triggering an integrated stress response dependent on ATF4 activation by specific eIF2α kinases. *Sci Signal*. 2016;9(415):ra18.
135~~4~~. Graves PR, et al. Mitochondrial protease ClpP is a target for the anticancer compounds ONC201 and related analogues. *ACS Chem Biol*. 2019;14(5):1020–1029.
136~~5~~. Ishizawa J, et al. Mitochondrial ClpP-mediated proteolysis induces selective cancer cell lethality. *Cancer Cell*. 2019;35(5):721–737.
137~~6~~. Baker TA, Sauer RT. ClpXP, an ATP-powered unfolding and protein-degradation machine. *Biochim Biophys Acta*. 2012;1823(1):15–28.
138~~7~~. Ishizawa J, et al. ATF4 induction through an atypical integrated stress response to ONC201 triggers p53-independent apoptosis in hematological malignancies. *Sci Signal*. 2016;9(415):ra17.
139~~8~~. Yuan X, et al. ONC201 activates ER stress to inhibit the growth of triple-negative breast cancer cells. *Oncotarget*. 2017;8(13):21626–21638.
140~~39~~. Ni X, et al. ONC201 selectively induces apoptosis in cutaneous T-cell lymphoma cells via activating pro-apoptotic integrated stress response and inactivating JAK/STAT and NF-κB pathways. *Oncotarget*. 2017;8(37):61761–61776.
141~~0~~. Fricker LD. Proteasome inhibitor drugs. *Annu Rev Pharmacol Toxicol*. 2020;60:457–476.
142~~1~~. Nalepa G, et al. Drug discovery in the ubiquitin-proteasome system. *Nat Rev Drug Discov*. 2006;5(7):596–613.
143~~2~~. Kyle RA, Rajkumar SV. Multiple myeloma. *Blood*. 2008;111(6):2962–2972.
144~~3~~. Manasanch EE, Orlowski RZ. Proteasome inhibitors in cancer therapy. *Nat Rev Clin Oncol*. 2017;14(7):417–433.
145~~4~~. Scott K, et al. Bortezomib for the treatment of multiple myeloma. *Cochrane Database Syst Rev*. 2016;4(4):CD010816.
146~~5~~. Kuhn DJ, et al. Potent activity of carfilzomib, a novel, irreversible inhibitor of the ubiquitin-proteasome pathway, against preclinical models of multiple myeloma. *Blood*. 2007;110(9):3281–3290.
147~~6~~. Hideshima T, et al. Small-molecule inhibition of proteasome and aggresome function induces synergistic antitumor activity in multiple myeloma. *Proc Natl Acad Sci U S A*. 2005;102(24):8567–8572.
148~~7~~. Ri M. Endoplasmic-reticulum stress pathway-associated mechanisms of action of proteasome inhibitors in multiple myeloma. *Int J Hematol*. 2016;104(3):273–280.
149~~8~~. Orlowski RZ. Why proteasome inhibitors cannot ERADicate multiple myeloma. *Cancer Cell*. 2013;24(3):275–277.
150~~49~~. Kawaguchi Y, et al. The deacetylase HDAC6 regulates aggresome formation and cell viability in response to misfolded protein stress. *Cell*. 2003;115(6):727–738.
151~~0~~. Cheng T, et al. Expression of histone deacetylase (HDAC) family members in bortezomib-refractory multiple myeloma and modulation by panobinostat. *Cancer Drug Resist*. 2021;4(4):888–902.
152~~1~~. Harada T, et al. Histone deacetylase inhibitors in multiple myeloma: from bench to bedside. *Int J Hematol*. 2016;104(3):300–309.
153~~2~~. Kikuchi S, et al. Class IIa HDAC inhibition enhances ER stress-mediated cell death in multiple myeloma. *Leukemia*. 2015;29(9):1918–1927.
154~~3~~. Zhang P, et al. HDAC4 protects cells from ER stress induced apoptosis through interaction with ATF4. *Cell Signal*. 2014;26(3):556–563.
155~~4~~. Hanke NT, et al. Carfilzomib combined with suberanilohydroxamic acid (SAHA) synergistically promotes endoplasmic reticulum stress in non-small cell lung cancer cell lines. *J Cancer Res Clin Oncol*. 2016;142(3):549–560.
156~~5~~. Baniulyte G, et al. Shared gene targets of the ATF4 and p53 transcriptional networks. *Mol Cell Biol*. 2023;43(8):426–449.
157~~6~~. Andrysik Z, et al. Identification of a core TP53 transcriptional program with highly distributed tumor suppressive activity. *Genome Res*. 2017;27(10):1645–1657.
158~~7~~. De Gassart A, et al. An inhibitor of HIV-1 protease modulates constitutive eIF2α dephosphorylation to trigger a specific integrated stress response. *Proc Natl Acad Sci U S A*. 2016;113(2):E117–E126.
